# Major vault protein (MVP) suppresses aging- and estrogen deficiency-related bone loss through Fas-mediated apoptosis in osteoclasts

**DOI:** 10.1038/s41419-023-05928-4

**Published:** 2023-09-13

**Authors:** Ruobing Wang, Yan Yang, Zhongyin Zhang, Na Zhao, Erik A. C. Wiemer, Jingjing Ben, Junqing Ma, Lichan Yuan

**Affiliations:** 1 Jiangsu Key Laboratory of Oral Diseases, Nanjing Medical University, Nanjing, China; 2 Department of Orthodontics, Affiliated Hospital of Stomatology, Nanjing Medical University, Nanjing, China; 3 Department of Medical Oncology, Erasmus MC Cancer Institute, University Medical Center Rotterdam, Rotterdam, the Netherlands; 4 Department of Pathophysiology, Key Laboratory of Targeted Intervention of Cardiovascular Disease, Collaborative Innovation Center for Cardiovascular Disease Translational Medicine, Nanjing Medical University, Nanjing, China

**Keywords:** Apoptosis, Bone

## Abstract

Osteoclasts (OCs), derived from monocyte/macrophage lineage, are key orchestrators in bone remodeling. Targeting osteoclast apoptosis is a promising approach to cut down excessive osteoclast numbers, and thus slow down the rate of bone mass loss that inevitably occurs during aging. However, the therapeutic target of apoptosis in osteoclasts has not been fully studied. Our previous work generated *Mvp*^*f/f*^*Lyz2-Cre* mice, conditionally depleting major vault protein (MVP) in monocyte lineage, and identified MVP as a bone protector for its negative role in osteoclastogenesis in vivo and in vitro. Here, we observed a notable decline of MVP in osteoclasts with aging in mice, encouraging us to further investigate the regulatory role of osteoclast MVP. Then, *Mvp*^*f/f*^*Lyz2-Cre* mice were exploited in two osteoporosis contexts, aging and abrupt loss of estrogen, and we revealed that conditional knockout of MVP inhibited osteoclast apoptosis in vivo and in vitro. Moreover, we reported the interaction between MVP and death receptor Fas, and MVP-Fas signaling cascade was identified to positively regulate the apoptosis of osteoclasts, thus preventing osteoporosis. Collectively, our comprehensive discovery of MVP’s regulatory role in osteoclasts provides new insight into osteoclast biology and therapeutic targets for osteoporosis.

## Introduction

Osteoporosis, one of the most common diseases of the elderly, currently stands as an emerging medical and socioeconomic threat as it increases the propensity of fragility fractures [1]. The unbalanced bone remodeling, specifically bone resorption over bone formation, leads to osteoporosis [2]. Epidemiological evidence and rodent studies concur that aging and abrupt loss of sex steroids, especially estrogen, are seminal culprits of osteoporosis [3]. Although the exact percentage of skeletal defects due to aging or estrogen loss has not been delineated, the current prevailing theory is that aging is the protagonist, and estrogen paucity accelerates the pace of aging-induced bone mass loss. Despite their different regulatory mechanisms concerning osteoporosis, they overlap in the number and life span of osteoclasts (OCs) in complex ways [4, 5]. Osteoclasts are key participants in orchestrating bone resorption by secreting acids and collagenolytic enzymes, and an excessive number of osteoclasts is responsible for bone loss in various diseases presenting osteoporotic phenotypes [6, 7]. Despite that multiple osteoclast-targeted therapies have shown promising results in osteoporosis treatment, the side effects cannot be overlooked. For instance, the ‘rebound effect’ following the discontinuation of denosumab, significantly compromised the clinical outcomes [1, 8, 9]. These limitations prompt us to further comprehensively characterize the biology of osteoclasts and identify new therapeutic targets.

Recently, targeting osteoclast apoptosis has been identified as a promising approach to cutting down excessive osteoclast numbers, and thus slowing down the rate of bone mass loss [10]. Among the various pathways involved in osteoclast apoptosis, Fas/FasL signaling pathway has gained much attention in the investigation of a therapeutic target for its intricate relationship with estrogen. Via estrogen receptor α (ERα), estrogen maintains bone mass by inducing FasL expression in osteoblasts to bind with Fas and shorten osteoclast lifespan [11–14]. The therapeutic activation or inhibition of Fas, however, has many side effects for its common expression [15]. Therefore, a target regulating Fas expression or function is needed. Pioneer studies have revealed the ubiquitous regulation of Fas in controlling cell numbers. The E3 ubiquitin ligase HRD1 was reported to protect B cells from activation-induced cell death by degrading the death receptor Fas [16]. In addition, cysteine-rich intestinal protein 1 (CRIP1) was established as a novel target for clinical drug resistance of colorectal cancer, which could interact with Fas and stimulate its ubiquitination and degradation [17]. However, how Fas expression is regulated in osteoclasts was ill-studied.

Major vault protein (MVP) has been increasingly appreciated as an influential regulator of intracellular signaling pathways [18, 19]. It is the main component of cellular ribonucleoprotein particles termed vault nanoparticles and is responsible for most functions of vaults, including diverse cellular signals and transport processes [20, 21]. For instance, MVP has been reported to interact with class A scavenger receptor (SR-A) and promote SR-A-mediated apoptosis in macrophages [22]. As MVP is highly conserved across species, it is rational to study the relationship between MVP and various human diseases using mouse models [20]. Previously, by exploiting MVP global knockout (*Mvp*^*−/−*^) mice and MVP monocyte-specific conditional knockout (*Mvp*^*f/f*^*Lyz2-Cre*) mice, we found that MVP deficiency resulted in osteoporotic phenotypes in vivo [23]. However, it is not fully elucidated how MVP is involved in the development of skeletal atrophy.

In the present study, we explored the effect of MVP in osteoclasts on bone remodeling in osteoporosis. MVP-Fas signaling cascade was identified to positively regulate osteoclast apoptosis, thus preventing osteoporosis. Our in-depth and comprehensive discovery of MVP would provide new insight into osteoclast biology and therapeutic targets for osteoporosis.

## Materials and methods

### Mice

All mice used during this study were housed under standard conditions in the animal facility of Nanjing Medical University. *Mvp*^*flox/flox*^(*C57BL/6*-*Mvp*^*em1(flox)Smoc*^) mice and *Lyz2-Cre* mice (*B6.129P2-Lyz*^*2tm1(cre)Ifo*^*/J*) were provided by the Key Laboratory of Targeted Intervention of Cardiovascular Disease, Nanjing Medical University (Nanjing, China). By crossing *Mvp*^*flox/flox*^ mice and *Lyz2-Cre* mice, MVP knockout mice specific to myeloid-specific cells were generated, which are termed *Mvp*^*flox/flox*^*Lyz2-Cre* mice [23]. Mice containing the double-floxed *Mvp* allele without the *Cre* recombinase gene were used as controls. Study protocols were approved by the Ethics Committee of the Stomatological School of Nanjing Medical University. All animal studies were performed under the guidelines of the Experimental Animal Care and Use Committee of Nanjing Medical University.

### Animal models

Mice were randomly allocated to experiment groups. In aging-related studies, 18-month-old C57BL/6 mice, *Mvp*^*f/f*^ mice, and *Mvp*^*f/f*^*Lyz2-Cre* mice of mixed genders were classified as old groups, and their 3-month-old counterparts were classified as young groups. Bilateral ovariectomy (OVX) was performed on 3-month-old *Mvp*^*f/f*^ and *Mvp*^*f/f*^*Lyz2-Cre* female mice under pentobarbital sodium anesthesia to abruptly terminate estrogen production and establish osteoporosis [13]. For sham groups, the ovaries were not resected but other surgical processes were identical to OVX groups. Two months after the operation, mice were euthanized for the following studies. There were no animal exclusion criteria. No blinding methods were used for surgeries.

### Immunofluorescence

After euthanasia, mouse femurs were isolated and fixed in 4% paraformaldehyde (PFA) at 4 °C overnight. Next, specimens were decalcified in 10% EDTA, dehydrated, embedded in paraffin, and sectioned into 4.5 μm thick slices. Sections were permeabilized in PBS containing 1% Triton X-100, blocked in normal goat serum for 30 min at 37 °C and incubated with primary antibodies against MVP (1:100, sc-18701, Santa Cruz, Texas, America), CTSK (1:100, sc-48353, Santa Cruz) and Cleaved-Caspases3 (1:100, #9664, Cell Signaling Technology, Massachusetts, America) 4 °C overnight. On the second day, sections were incubated with secondary antibodies for 1 h at 37 °C and finally stained with 4’,6-diamidino-2- phenylindole (DAPI) (Beyotime, Shanghai, China) for 2 min at room temperature.

For in vitro studies, osteoclasts were harvested in 4% PFA for 15 min at room temperature, permeabilized with 0.5% Triton X-100 and blocked with normal goat serum at 37 °C for 1 h. Then, cells were co-stained with primary antibodies against MVP (1:100, sc-18701, Santa Cruz) and Fas (1:100, sc-74540, Santa Cruz) at 4 °C overnight. The next day, after washing with PBST, cells were incubated with secondary antibodies at 37 °C for 1 h. Nuclei were stained with DAPI. Images were captured using a fluorescence microscope (Leica Microsystems, Wetzlar, Germany).

### In vitro cell isolation and culture

After mice were euthanized, their femurs were isolated and bone marrow monocytes (BMMs) were flushed out using α-minimum essential medium (α-MEM) containing 2% FBS. After lysis of red blood cells, BMMs were immediately cultured in α-minimum essential medium (α-MEM) (Gibco, Massachusetts, America) containing 10% FBS (AusGeneX, Gold Coast, Australia), 1% penicillin/streptomycin (NCE Biotech, Suzhou, China), M-CSF (30 ng/mL, R&D, Minnesota, America) and RANKL (10 ng/mL, R&D). We previously reported that MVP expression hits its peak on the 5th day upon osteoclastogenesis induction. Therefore, cells were fixed by 4% paraformaldehyde (PFA) on day 5 for the following assays in this study [23].

### Western blot assay

Protein extraction of whole cells was performed as previously described [24]. Equivalent levels of protein were resolved by 10%, or 15% SDS–PAGE gel (E303-01 or E305-01, Vazyme, Nanjing, China), transferred onto polyvinylidene fluoride (PVDF) membranes (Millipore, Massachusetts, USA), and then blocked with 1x Protein Free Rapid Blocking Buffer (Epizyme, Shanghai, China). PVDF membranes were incubated with primary antibodies overnight at 4 °C, washed with TBST three times, incubated with secondary antibodies, and last visualized by enhanced chemiluminescence using Chemiluminescence gel imaging system (Tanon 5200, Tanon, Shanghai, China). Semi-quantification of the results was assessed by Image J software (National Institutes of Health, USA). Antibodies used are as follows: MVP (1:1000, sc-23916, Santa Cruz), GAPDH (1:2000, AF2819, Beyotime), Cleaved-Caspase3 (1:1000, #9664, Cell Signaling Technology), Cleaved-Caspase8 (1:1000, #8592, Cell Signaling Technology), Bax (1:1000, #T40051, Abmart, Shanghai, China), Bcl-2 (1:1000, #T40056, Abmart), Fas (1:1000, sc-74540, Santa Cruz), ERα (1:1000, sc-8005, Santa Cruz), and Ubiquitin (1:1000, #3936, Cell Signaling Technology). Original western blots are included in **Supplementary Material**.

### Quantitative real-time PCR (qRT -PCR)

Total cellular RNA was isolated by FastPure Cell/Tissue Total RNA Isolation Kit V2 (RC112-01, Vazyme) and then reverse-transcribed into cDNA using HiScript II Q RT SuperMix for qPCR (R222-01, Vazyme) per manufacturer instructions. qRT-PCR was performed using ChamQ SYBR qPCR Master Mix (Q341-02/03, Vazyme), and conducted on QuantStudio 7 Pro Real-Time PCR System (Applied Biosystems, Massachusetts, USA). Primers used in this study are included in Table S1.

### In vitro TRAP staining

After cell fixation on the 5th day of osteoclastic induction, osteoclast numbers were assessed following the manufacturer’s instructions for Leukocyte Acid Phosphatase (TRAP) kit (387A-1KT, Sigma-Aldrich, Munich, America). TRAP-positive multinucleated cells with 3 or more nuclei were counted as osteoclasts. Images were visualized under a light microscope (Leica Microsystems).

### In vitro TUNEL assay

After 5 days of osteoclastogenesis induction, osteoclast apoptosis in vitro was examined using the In-Situ Cell Death Detection Kit following the manufacturer’s instructions (#12156792910, Roche, Rotkreuz, Switzerland). Images were visualized under a fluorescence microscope (Leica Microsystems).

### Micro-computed tomography (μCT) analysis

Complete mouse femurs were separated mechanically from soft tissue and fixed in 4% PFA at 4 °C overnight. Specimens were scanned with a μCT scanner (vivaCT 80, SCANCO, Florida, Switzerland), set to 55 kV and 83 μA at 15.6 μm resolution, and then analyzed using CTAn v1.13.8.1 as previously described [25]. The following parameters were analyzed: trabecular bone volume per total volume (BV/TV), trabecular number (Tb.N), trabecular thickness (Tb.Th), trabecular separation (Tb.Sp), and cortical porosity.

### Histological analysis

After scanning, isolated mouse femurs were decalcified in 10% EDTA, dehydrated, embedded in paraffin, and sectioned into 4.5 μm thick slices. Hematoxylin and eosin (H&E) staining and TRAP staining were used to examine the bone quality as previously described [23]. TRAP-positive multinucleated cells with 3 or more nuclei were counted as osteoclasts. Images were visualized under a light microscope (Leica Microsystems).

### Lentiviral transfection

BMMs were prepared and infected with control or MVP-overexpressing lentiviruses (GeneChem, Shanghai, China) at a multiplicity of infection (MOI) of 30 with the help of 5 μg/mL polybrene. After 16 h of transfection, cells were rinsed twice with PBS and cultured in the fresh medium of osteoclastic induction for 5 days.

### Immunoprecipitation (IP) and ubiquitination assay

Cells were lysed in IP lysis buffer (P10013, Beyotime) containing protease inhibitor cocktail tablets for 15 min at 4 °C. After the protein concentrations were measured, equal levels of lysates were used for immunoprecipitation. Cell lysates were incubated with antibodies against MVP (sc-23916, Santa Cruz) or Fas (sc-74540, Santa Cruz) and protein A/G beads (sc-2003, Santa Cruz) overnight at 4 °C. Then, the precipitants were washed three times with IP lysis buffer, and the immune complexes were eluted with 2x loading buffer for 5 min at 95 °C, and subjected to western blot analysis. For ubiquitination assay, 4 h before cell harvest, 10 µΜ MG-132 (MCE, New Jersey, USA) was added into the medium.

### AAV-Mvp treatment

Ovariectomy (OVX) or sham surgery was performed on 3-month-old *Mvp*^*f/f*^*Lyz2-Cre* female mice. One week and two weeks after the surgery, mice were subjected to a local femur injection of 5 µL AAV (titer = 10^12-13 ^v.g./mL, GeneChem) expressing GFP with or without MVP gene [26]. Mice were sacrificed after 8 weeks of OVX surgery. Femurs were then isolated, and fixed in 4% PFA at 4 °C overnight for the following examinations. No blinding methods were used for injections.

### Immunohistochemical (IHC) staining

After complete dewaxing, sections were boiled in sodium citrate buffer solution to retrieve antigens and quenched with 3% hydrogen peroxide (H_2_O_2_). Normal goat serum was used to block tissues at 37 °C for 30 min, followed by incubation with primary antibodies overnight at 4 °C: anti-MVP (1:100, sc-23916, Santa Cruz). The next day, sections were incubated with secondary antibodies and visualized using a diaminobenzidine (DAB) kit (DAB-0031, MaxVision, Fuzhou, China) [25]. Cell nuclei were counterstained with hematoxylin. Images were captured under a light microscope (Leica Microsystems).

### scRNA-seq data analysis

The scRNA-seq data of the primary human femoral head tissue cells were retrieved from Gene Expression Omnibus database at GSE169396. The R package Seurat (V4.2.1) was utilized for analysis. Quality control was performed with the default settings. Significantly available dimensions were identified by PCA with the criteria of *P* < 0.05, and 20 initial principal components were applied to dimension reduction using t-Distributed Stochastic Neighbor Embedding (t-SNE). Cell types annotations were conducted according to the primary report [27].

### Statistics

No statistical methods were used to predetermine the sample size. All experiments were repeated three times in this study. Data are expressed as mean ± SD. The significance of differences between the two groups was determined by a two-tailed Student’s unpaired *t* test. Statistical comparisons of more than two groups were performed using One-way ANOVA followed by Dunnett’s post-hoc comparisons. All results were analyzed using Prism 9.0 statistical software (GraphPad, California, America). *P* value < 0.05 was considered significant. *P < 0.05 and **P < 0.01.

## Results

### MVP expression is downregulated in osteoclasts from old mice

**Fig. 1:**
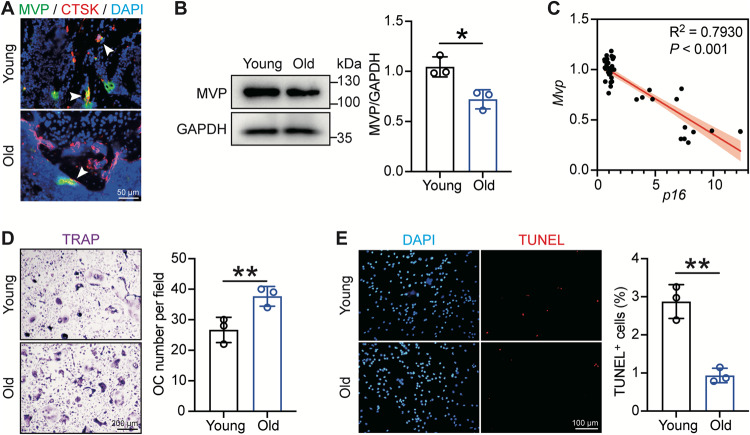
Osteoclasts from aged mice exhibit reductions in MVP expression and apoptosis. **A** Paraffin sections of distal femurs from young (3 months) and old (18 months) C57BL/6 mice were subjected to immunofluorescence staining with antibodies against MVP (green) and CTSK (red). Nuclei were stained with DAPI and appeared blue. White arrows indicate CTSK^+^/MVP^+^ cells. **B** BMMs isolated from young (3 months) and old (18 months) C57BL/6 mice were cultured in the presence of M-CSF (30 ng/mL) and RANKL (10 ng/mL). After 5 days of osteoclastogenesis induction, MVP protein levels were detected by western blot and assessed by densitometric analysis (*n* = 3). **C** Correlation between *p16* and *Mvp* mRNA levels of young-mouse-origin and old-mouse-origin osteoclasts, detected by qRT-PCR, were analyzed using single linear regression. **D** TRAP staining was performed after 5 days of osteoclastic induction. TRAP^+^ cells with three or more nuclei were counted as osteoclasts and were scored per field (*n* = 3). **E** Apoptosis in osteoclasts was determined by TUNEL cell death detection kit and apoptotic cells appeared bright red. Nuclei were stained with DAPI and appeared blue. Representative images and quantification of apoptosis percentage are shown (*n* = 3). Data are mean ± SD and were analyzed by a two-tailed Student’s unpaired *t* test. *P < 0.05 and **P < 0.01.

To determine the role of osteoclast MVP in aging-induced osteoporosis, we first detected its expression in mouse models. As shown in Fig. 1A, there were more CTSK^+^ cells (osteoclast specific marker) lying on the skeletal inner surface in old wildtype C57BL/6 mice (18 months) compared with the young group (3 months), while MVP^+^/CTSK^+^ cells were fewer. To explore the aging-related MVP expression alterations in osteoclasts, bone marrow monocytes (BMMs) were isolated from young and old mice and stimulated into osteoclasts in the presence of M-CSF and RANKL [28, 29]. We found a remarkable decline in the protein level of MVP in old-mouse-origin osteoclasts compared with young-mouse-origin osteoclasts (Fig. 1B). In addition, single-linear regression showed a robust negative correlation between *Mvp* and the senescence marker *p16* on mRNA levels in osteoclasts. (Fig. 1C). Moreover, in line with previous studies, although the osteoclast levels were significantly higher in the elderly group assessed by osteoclast-specific TRAP staining, their apoptotic activities were profoundly attenuated compared with the young group [30, 31] (Fig. 1D, E). Osteoclasts are terminally differentiated cells, and their numbers are orchestrated by osteoclastogenesis from monocyte/macrophage lineage and cell death, mainly apoptosis [6]. Our previous study identified MVP as a mediator negatively regulating osteoclastogenesis in the bone microenvironment. Here, we identified aging-induced MVP reduction as well as attenuated apoptosis in osteoclasts. Consequently, these results encouraged us to explore the relationship between MVP and apoptosis in osteoclasts and its role in the pathogenesis of osteoporosis.

### MVP depletion augments aging-induced bone mass loss and reduces osteoclast apoptosis in vivo

**Fig. 2:**
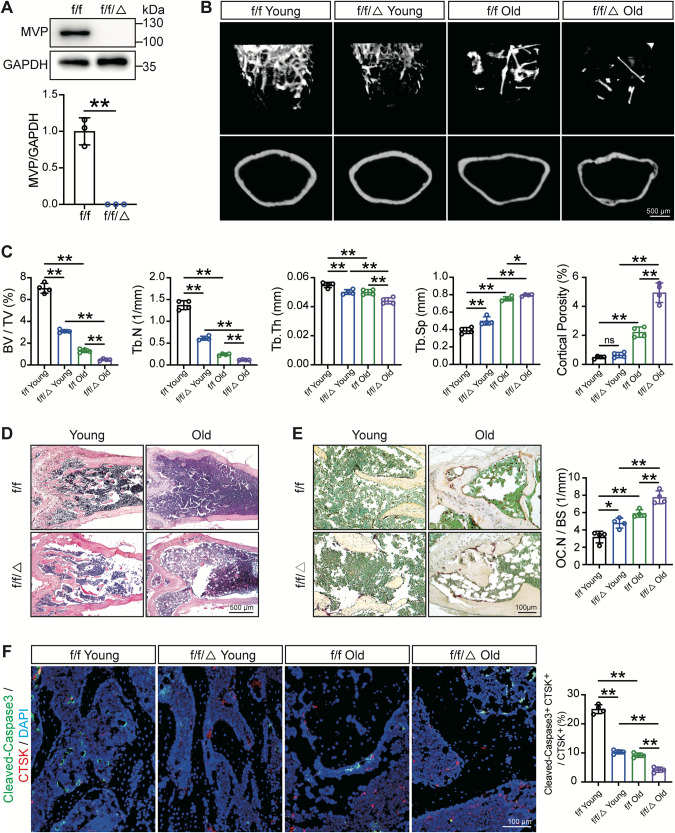
MVP depletion augments aging-induced bone mass loss and reduces osteoclast apoptosis in vivo. **A** MVP expression and quantification of osteoclasts determined by western blot from 8-week-old *Mvp*^*f/f*^ and *Mvp*^*f/f*^*Lyz2-Cre* mice (*n* = 3). **B** Representative µCT reconstruction of the cancellous bone and the cortical bone of distal femurs from 3-month-old or 18-month-old *Mvp*^*f/f*^ and *Mvp*^*f/f*^*Lyz2-Cre* mice (*n* = 4). **C** Bone morphometric parameters of the cancellous bone and the cortical bone of distal femurs from 3-month-old or 18-month-old *Mvp*^*f/f*^ and *Mvp*^*f/f*^*Lyz2-Cre* mice. BV/TV, bone volume/tissue volume; Tb.N, trabecular number; Tb.Th, trabecular thickness; Tb.Sp, trabecular separation (*n* = 4). **D** Representative H&E staining of distal femurs in the indicated groups (*n* = 4). **E** Representative TRAP staining of the cancellous bone from 3-month-old or 18-month-old *Mvp*^*f/f*^ and *Mvp*^*f/f*^*Lyz2-Cre* mice with quantification of osteoclast numbers (*n* = 4). **F** Representative immunofluorescence staining with Cleaved-Caspase3 (green) and CTSK (red) antibodies of distal femurs and quantification in the indicated groups (*n* = 4). Data are mean ± SD and were analyzed by One-way ANOVA followed by Dunnett’s post-hoc comparisons. *P < 0.05 and **P < 0.01.

To further investigate MVP’s role in regulating osteoporosis in vivo, we used previously established *Mvp*^*f/f*^*Lyz2-Cre* mouse models, which exhibited osteoporotic phenotypes compared to *Mvp*^*f/f*^ or *Lyz2-Cre* mice. The knockout of MVP in osteoclasts was confirmed by western blot (Fig. 2A). Firstly, micro-computed tomography (µCT) was used to detect bone mass and micro-structures in the aging mouse models. As shown in Fig. 2B and Fig. 2C, 18-month-old *Mvp*^*f/f*^*Lyz2-Cre* mice showed severer osteoporotic phenotypes in comparison with 18-month-old *Mvp*^*f/f*^ mice, as evidenced by multiple parameters. Specifically, 18-month-old *Mvp*^*f/f*^*Lyz2-Cre* mice had a less trabecular number, thinner trabecular thickness, and larger trabecular separation in distal femurs compared to 18-month-old *Mvp*^*f/f*^ mice [32]. Meanwhile, concerning cortical bone, there is no significant difference in porosity between 3-month-old *Mvp*^*f/f*^ and *Mvp*^*f/f*^*Lyz2-Cre* mice, while the percentage of cortical porosity in 18-month-old *Mvp*^*f/f*^*Lyz2-Cre* mice was twofold of it in their age-matched counterparts. Complementarily, H&E staining showed similar results (Fig. 2D). These results indicated that MVP might play as a bone protector in aging-related osteoporosis.

To investigate whether conditional knockout of MVP increases the number of osteoclasts aggravating bone loss in the osteoporotic context, we next assessed the relationship between osteoclast numbers and MVP. As shown in Fig. 2E, there were more osteoclasts around the cancellous bone of distal femurs of 18-month-old *Mvp*^*f/f*^*Lyz2-Cre* mice compared to age-matched *Mvp*^*f/f*^ mice. Given that targeting apoptosis is an instrumental approach to regulating the number of osteoclasts, we hypothesized that the depletion of MVP inhibits osteoclast apoptosis, thus presenting more osteoclasts. To address this issue, we performed immunofluorescence staining with antibodies of the apoptotic marker Cleaved-Caspase3 and CTSK on distal femurs. We found that 3-month-old *Mvp*^*f/f*^*Lyz2-Cre* mice displayed less apoptosis in osteoclasts (CTSK^+^/Cleaved-Caspase3^+^) compared to 3-month-old *Mvp*^*f/f*^ mice. In addition, 18-month-old *Mvp*^*f/f*^*Lyz2-Cre* mice showed less apoptosis in osteoclasts compared to their age-matched *Mvp*^*f/f*^ mice (Fig. 2F). Taken together, MVP may control osteoclast numbers by promoting osteoclast apoptosis in aging-related osteoporosis.

### MVP depletion augments OVX-induced bone mass loss and reduces osteoclast apoptosis in vivo

**Fig. 3:**
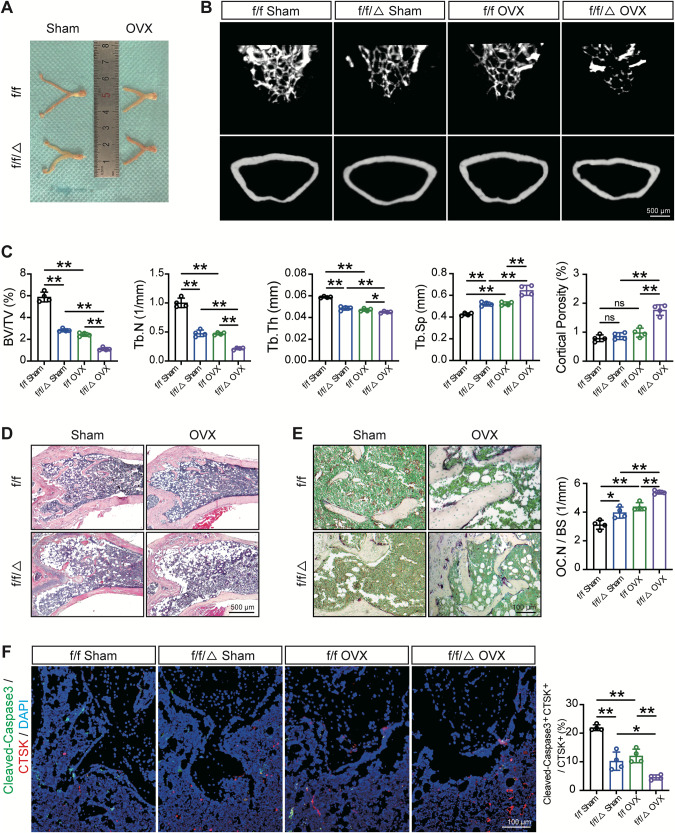
MVP depletion augments OVX-induced bone mass loss and reduces osteoclast apoptosis in vivo. **A** 3-month-old female *Mvp*^*f/f*^ and *Mvp*^*f/f*^*Lyz2-Cre* mice were operated on sham surgery or OVX, and sacrificed 2 months after the surgery. Mouse uteri size of *Mvp*^*f/f*^ and *Mvp*^*f/f*^*Lyz2-Cre* mice after euthanasia (*n* = 4). **B** Representative µCT reconstruction images of the cancellous bone and the cortical bone of distal femurs in indicated groups (*n* = 4). **C** Morphometric parameters of the cancellous bone and the cortical bone in distal femurs (*n* = 4). **D** Representative H&E staining of distal femurs in the indicated groups (*n* = 4). **E** Representative TRAP staining of the cancellous bone of *Mvp*^*f/f*^ and *Mvp*^*f/f*^*Lyz2-Cre* mice operated on sham surgery or OVX osteoclast numbers with quantification (*n* = 4). **F** Representative immunofluorescence staining with Cleaved-Caspase3 (green) and CTSK (red) antibodies of distal femurs and quantification of *Mvp*^*f/f*^ and *Mvp*^*f/f*^*Lyz2-Cre* mice operated on sham surgery or OVX (*n* = 4). Data are mean ± SD and were analyzed by One-way ANOVA followed by Dunnett’s post-hoc comparisons. *P < 0.05 and **P < 0.01.

Given that high levels of osteoclast-mediated bone resorption are not only due to advanced age but also to the abrupt loss of sexual steroids, especially estrogen [4], we next examined whether depletion of osteoclast MVP plays a role in estrogen deficiency-induced osteoporosis. Both the *Mvp*^*f/f*^ and *Mvp*^*f/f*^*Lyz2-Cre* female mice were operated on sham surgery or ovariectomy (OVX) at 3 months old and their femurs were scanned by µCT 2 months after the surgery. Successful OVX was evidenced by uterine size (Fig. 3A). Reconstruction of µCT results showed that OVX dramatically reduced cancellous bone mass in both *Mvp*^*f/f*^ and *Mvp*^*f/f*^*Lyz2-Cre* mice, by 58% and 61% of BV/TV respectively. Additionally, parameters including Tb.N, Tb.Th markedly declined while Tb.Sp dramatically increased in post-OVX *Mvp*^*f/f*^*Lyz2-Cre* mice compared to post-OVX *Mvp*^*f/f*^ mice. What’s more, cortical porosity was reduced by nearly 61% in the post-OVX *Mvp*^*f/f*^*Lyz2-Cre* group compared to the post-OVX *Mvp*^*f/f*^ mice (Fig. 3B, C). Similar results were observed by H&E analysis (Fig. 3D). Moreover, post-OVX *Mvp*^*f/f*^*Lyz2-Cre* mice showed more osteoclasts compared to their *Mvp*^*f/f*^ counterparts (Fig. 3E). Complementarily, there was fewer osteoclast apoptosis in *Mvp*^*f/f*^*Lyz2-Cre* mice operated OVX in comparison with their *Mvp*^*f/f*^ counterparts (Fig. 3F). Taken together, we showed that MVP knockout inhibited osteoclast apoptosis, thus contributing to aging- or OVX-related bone loss.

### MVP promotes apoptosis of osteoclasts in vitro

**Fig. 4:**
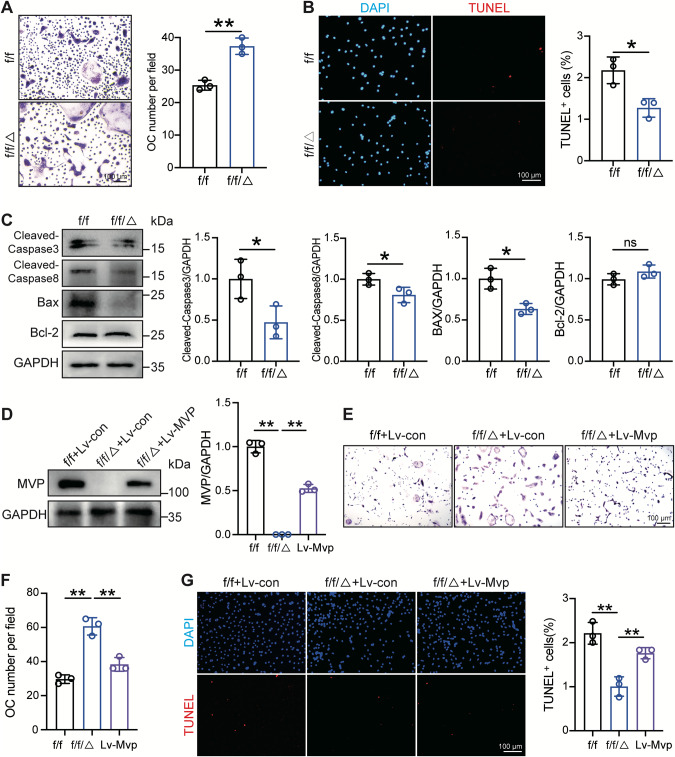
MVP promotes osteoclast apoptosis in vitro. **A** BMMs were isolated from the femoral bone marrow of 8-week-old *Mvp*^*f/f*^ and *Mvp*^*f/f*^*Lyz2-Cre* mice and induced into mature osteoclasts with M-CSF and RANKL. Representative images and quantification of osteoclast numbers assessed by TRAP staining (*n* = 3). **B** Representative images of TUNEL staining (red) and DAPI (blue) of osteoclasts from *Mvp*^*f/f*^ and *Mvp*^*f/f*^*Lyz2-Cre* mice with quantification of apoptosis percentage (*n* = 3). **C** Western blot and quantification showing levels of apoptosis-related proteins in *Mvp*^*f/f*^ and *Mvp*^*f/f*^*Lyz2-Cre* osteoclasts (*n* = 3). **D** Western blot and quantification of MVP expression in osteoclasts from the femoral bone marrow of 8-week-old *Mvp*^*f/f*^ and *Mvp*^*f/f*^*Lyz2-Cre*, transfected with Lv-con or Lv-Mvp for 16 hours and then cultured in osteoclastogenesis medium for 5 days (*n* = 3). **E**, **F** Representative images and quantification of osteoclasts of indicated groups using TRAP staining (*n* = 3). **G** Representative images and quantification of TUNEL staining (red) and DAPI (blue) of osteoclasts from *Mvp*^*f/f*^ and *Mvp*^*f/f*^*Lyz2-Cre* mice (*n* = 3). Data are mean ± SD and were analyzed by One-way ANOVA followed by Dunnett’s post-hoc comparisons. *P < 0.05 and **P < 0.01.

After observing attenuated apoptosis of osteoclasts in vivo, we next examined the effects of MVP depletion on apoptosis in vitro. As shown in Fig. 4A and Fig. 4B, MVP depletion increased osteoclast numbers while inhibiting osteoclast apoptosis. Concordantly, western blot results showed decreases in Cleaved-Caspase3, Cleaved-Caspase8, and Bax, and a negligible alteration in Bcl-2 (Fig. 4C). Taken together, these results unveiled the pro-apoptotic role of MVP in osteoclasts. To further clarify this function, we exploited Lv-Mvp to re-generate MVP in *Mvp*^*f/f*^*Lyz2-Cre* osteoclasts. Successful lentiviral infection was evidenced by green fluorescent protein (GFP) expression (Fig. S1A) and western blot (Fig. 4D). TRAP staining assay showed attenuated osteoclast numbers in Lv-Mvp transfected *Mvp*^*f/f*^*Lyz2-Cre* osteoclasts (Fig. 4E, F). In parallel, we found that the re-expression of MVP in *Mvp*^*f/f*^*Lyz2-Cre* osteoclasts partially reversed the reduction of osteoclast apoptosis induced by MVP knockout (Fig. 4G). Collectively, these results suggested that MVP might regulate osteoclast levels by promoting apoptosis.

### MVP inhibits Fas ubiquitination to promote osteoclast apoptosis

**Fig. 5:**
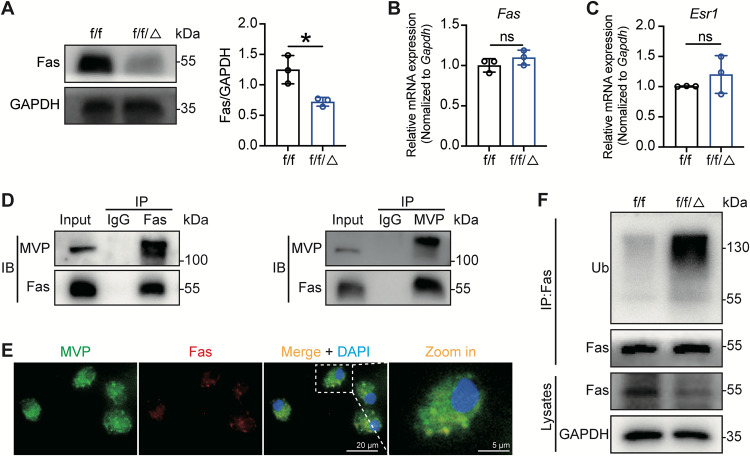
MVP interacts with Fas and inhibits its ubiquitination in osteoclasts. **A** Western blot showing Fas protein levels relative to GAPDH from osteoclasts isolated from 8-week-old *Mvp*^*f/f*^ and *Mvp*^*f/f*^*Lyz2-Cre* mice following 5 days of osteoclastogenesis induction (*n* = 3). **B**, **C** qRT-PCR results of *Fas* and *Esr1* in osteoclasts originated from *Mvp*^*f/f*^ and *Mvp*^*f/f*^*Lyz2-Cre* mice (*n* = 3). **D** Osteoclasts, isolated from 8-week-old wild-type mice, were harvested on the 5th day of osteoclastogenic induction. Whole-cell lysates were subjected to immunoprecipitation with anti-Fas (left panels) or anti-MVP (right panels) antibodies followed by immunoblot with antibodies against Fas and MVP. **E** Cellular immunofluorescence assay showed Fas (red) co-localized with MVP (green) in BMMs cultured in osteoclastogenesis medium for 5 days. Nuclei were stained with DAPI (blue). **F** Following 5 days of osteoclastogenic induction and 4 hours of incubation by MG-132, the whole cell lysates of *Mvp*^*f/f*^ and *Mvp*^*f/f*^*Lyz2-Cre* osteoclasts were subjected to immunoprecipitation with poly-ubiquitin antibody and the precipitants were performed by western blot to show the ubiquitin level of Fas. Data are mean ± SD and were analyzed by a two-tailed Student’s unpaired *t* test. *P < 0.05 and **P < 0.01.

Given that Fas-mediated apoptosis potently regulates the level of osteoclasts, which is correlated with aging and estrogen, we next sought to investigate the relationship between MVP and Fas-mediated apoptosis. As shown in Fig. 5A, we found that MVP depletion decreased the protein level of Fas. Nevertheless, the *Fas* mRNA level remained unchanged (Fig. 5B). In addition, qRT-PCR and western blot showed no significant alterations in *Esr1* and ERα expressions between *Mvp*^*f/f*^*Lyz2-Cre* and *Mvp*^*f/f*^ osteoclasts (Fig. 5C, S2A), excluding the difference of estrogen sensitivity between two groups. These results encouraged us to explore whether MVP ablation decreased Fas expression on the post-transcriptional level. Reciprocal immunoprecipitation (IP) assays detected interactions between endogenous MVP and Fas in wildtype osteoclasts (Fig. 5D). In concordance, immunofluorescence staining assay showed that MVP and Fas were mainly co-localized in the cytoplasm of osteoclasts (Fig. 5E). Given that ubiquitination plays an important role in regulating Fas-mediated apoptosis [33, 34], we wondered whether MVP could stabilize Fas. IP assay showed that compared with the *Mvp*^*f/f*^ group, osteoclasts from *Mvp*^*f/f*^*Lyz2-Cre* mice displayed more ubiquitin attached to Fas (Fig. 5F). To sum up, these results suggested that MVP stabilizes Fas by inhibiting its ubiquitination, thus promoting osteoclast apoptosis.

### MVP re-expression rescues OVX-induced bone mass loss and promotes osteoclast apoptosis in vivo

**Fig. 6:**
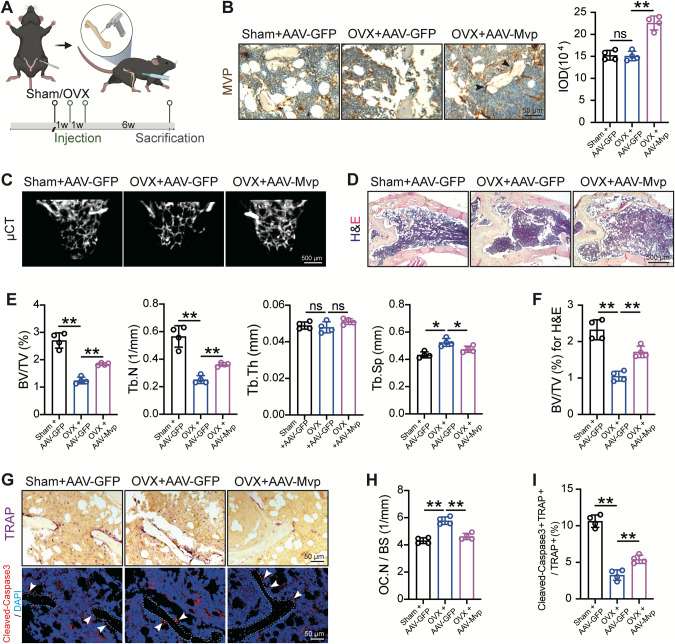
MVP re-expression attenuates the reduction of bone mass and osteoclast apoptosis induced by OVX in vivo. **A** Schematic diagram of mouse models. 3-month-old *Mvp*^*f/f*^*Lyz2-Cre* mice were operated on sham surgery or OVX and were subjected to local injection of AAV-GFP or AAV-Mvp in femurs twice. Mice were sacrificed 6 weeks after the last injection. **B** Representative IHC staining of MVP (black arrow) around the cancellous bone with quantification (*n* = 4). **C**, **E** Representative µCT reconstruction of the cancellous bone from distal femurs of indicated groups with quantifications (*n* = 4). **D**, **F** Representative HE staining images and quantification of distal femurs (*n* = 4). **G** Representative TRAP staining images and representative immunofluorescence images using anti-Cleaved-Caspase3 (red) of the same region around cancellous bones (white dotted line). Nuclei were stained with DAPI and appeared blue. White arrows indicate Cleaved-Caspase3^+^TRAP^+^ cells. **H** Quantifications of osteoclast numbers of **G** (*n* = 4). **I** Quantifications of the Cleaved-Caspase3^+^TRAP^+^/ TRAP^+^ percentage of **G** (*n* = 4). Data are mean ± SD and were analyzed by One-way ANOVA followed by Dunnett’s post-hoc comparisons. *P < 0.05 and **P < 0.01.

Next, we set out to verify the clinical potential of MVP in preventing osteoporosis by employing *Mvp*^*f/f*^*Lyz2-Cre* mouse models with OVX surgery. Successful elimination of bi-literal ovaries was evidenced by uterine size (Fig. S3A). As illustrated in Fig. 6A, adeno-associated virus (AAV) was locally injected for ectopic MVP expression in mouse femurs. GFP expression (Fig. S3B) as well as IHC staining of MVP (Fig. 6B) proved the effectiveness of AAV. In addition, reconstruction of µCT results revealed that OVX significantly reduced bone mass while forced ectopic MVP expression partially attenuated this destruction in cancellous bone, which was also validated by multiple parameters (Fig. 6C, E). H&E staining showed similar results (Fig. 6D, F). Moreover, the increase of osteoclasts in OVX models was neutralized by AAV-Mvp, in parallel with the elevation of apoptosis activity in cells lying on the surface of the cancellous bone (Fig. 6G-I). Taken together, our results indicated that MVP could be utilized as a novel target to prevent osteoporosis.

## Discussion

Here, we found that MVP suppressed bone resorption along with osteoclast numbers induced by aging or abrupt estrogen loss. Mechanically, MVP inhibited the ubiquitination of Fas in osteoclasts, which in turn increased osteoclast apoptosis. These results emphasize the role of osteoclast MVP in the regulation of osteoporosis.

We previously showed that MVP global knockout (*Mvp*^*-/-*^) mice or MVP monocyte-specific conditional knockout (*Mvp*^*f/f*^*Lyz2-Cre*) mice exhibited less bone mass and more osteoclast numbers compared to their wildtype counterparts. Although MVP expression in osteoblasts was validated in vivo (data not shown), the global MVP knockout did not affect the bone formation rate of 3-week-old mice. *Mvp*^*f/f*^*Lyz2-Cre* mice showed a similar bone formation rate to *Mvp*^*f/f*^ mice [23]. Therefore, our previous work focused on MVP in osteoclasts and identified the MVP-NFATc1 interaction in suppressing osteoclastogenesis.

Here, we found that MVP expression in osteoclasts of 18-month-old WT mice was reduced. Since aging and deficiency of estrogen are established etiologies of osteoporosis, which inexorably overlap in increasing osteoclast abundance, we utilized two different but complementary mouse models to illustrate the relationship between osteoclast MVP and osteoporosis. Results showed that MVP prevented aging- and OVX-related bone loss. Specifically, *Mvp*^*f/f*^*Lyz2-Cre* mice exhibited stronger apoptotic activity accompanied by severer deterioration of bone mass in these two osteoporotic contexts. Furthermore, forced ectopic MVP expression attenuated bone loss in OVX mouse models, verifying the regulatory role of MVP in the bone microenvironment and the translational potential of the present study.

Several studies have linked MVP to apoptosis in various cells, displaying contradictory roles of promoting or inhibiting apoptosis. For example, upon apoptotic stresses, MVP expression significantly increased in HDFs compared to young HDFs. Moreover, MVP knockdown markedly reduces the level of antiapoptotic protein Bcl-2, which might explain the resistance of apoptosis in senescent HDFs [35, 36]. However, in macrophages, which are precursors of osteoclasts, MVP functions as a pro-apoptotic mediator that interacts with SR-A and subsequently activates p38 and JNK signaling pathways to induce the apoptosis of macrophages [22]. In the present study, we reported that MVP reduced osteoclast numbers in vivo and in *vitro*.

Pioneering reports have documented the critical role of MVP in regulating diverse biological progresses through interacting with multiple proteins, including cell proliferation, cell survival, and transportation of drugs or proteins between the nucleus and cytoplasm [20]. For instance, MVP inhibits inflammatory response in macrophages through binding with TRAF6 and preventing its oligomerization and ubiquitination [37]. In addition, MVP interacts with IRF2 to destabilize the interaction between HDM2 and IRF2, which subsequently degrades p53 and results in tumorigenesis in hepatocellular carcinoma [38]. Based on previous studies characterizing the central role of Fas-FasL signaling in regulating osteoclast apoptosis, here, we found that Fas protein expression decreased in MVP knockout osteoclasts while the Fas mRNA remained stable. Further reciprocal IP and immunofluorescence experiments identified the interaction between MVP and Fas. Additionally, we observed that MVP knockout significantly elevated the ubiquitin level of Fas in osteoclasts. Given the protein-degradation role of ubiquitination, we proposed that MVP regulated bone homeostasis by inhibiting the ubiquitination of Fas in osteoclasts, which resulted in elevated apoptosis activities [16, 17]. The functional FasL is expressed in osteoblasts and initiates osteoclast apoptosis through Fas/FasL signaling [13, 39]. A further study investigating whether MVP regulated osteoblast-osteoclast communications in homeostatic or osteoporotic skeletal microenvironments is required.

Bone remodeling is a complex process constructed by the concerted efforts of a group of cells termed the basic multicellular unit (BMU). In addition to osteoclasts and osteoblasts, the BMU contains osteomacs, osteocytes, bone lining cells, and capillary blood supply [40]. To validate and explore the regulatory role of MVP in human bone remodeling, a public dataset GSE169396 was employed, which contains the single cell-RNA sequencing results from four human femoral head samples. After quality control, these cells were classified into 17 distinct clusters and then into 9 cell types (Fig. 4SA-C). MVP was mainly expressed in endothelial cells (ECs), plasmacytoid dendritic cells (pDCs), and monocytes (Fig. S4D). MVP distribution in pDCs ranked first, in part supporting the established role of MVP in immunology [20]. Based on our work, definitive proof of MVP’s role on osteoclasts (derived from one of the subtypes of monocytes) in human bone tissues requires additional studies. Moreover, recent evidence showed that the aging-related reduced blood flow might directly down-regulate the Notch signaling in ECs and subsequently contribute to aging-related bone loss by aberrant osteoblast vigor [41]. Whether MVP could affect ECs and thus affect aging-related osteoporosis warrants further study.

In summary, we identified MVP as a novel factor for Fas stability and elucidated the essential pro-apoptotic role of the MVP-Fas pathway in osteoclasts. Given the fact that osteoclast MVP is downregulated with aging, MVP offers a potent target to treat the most common bone disease in the elderly.

## Supplementary information

Supplementary Tables and Figure Legends

Supplementary Figure 1

Supplementary Figure 2

Supplementary Figure 3

Supplementary Figure 4

Original Data File

Reproducibility Checklist

## Data Availability

The datasets generated and/or analyzed during the current study are available from the corresponding author on reasonable request.
