# Supplementary material for: Major vault protein (MVP) suppresses aging- and estrogen deficiency-related bone loss through Fas-mediated apoptosis in osteoclasts
Source: Cell Death Dis. 2023 Sep 13;14(9):604. doi: 10.1038/s41419-023-05928-4 (PMC10500014; doi:10.1038/s41419-023-05928-4)
Supplement: Supplementary file 1 — Supplementary Tables and Figure Legends [file 41419_2023_5928_MOESM1_ESM.docx]

**SUPPLEMENTARY TABLES**

**Table S1** List of primer sequences used for the qRT-PCR analysis

| **Gene** | **Forward primer (5’-3’)** | **Reverse primer (5’-3’)** |
| --- | --- | --- |
| *Gapdh* | ACCACAGTCCATGCCATCAC | TCCACCACCCTGTTGCTGTA |
| *Mvp* | TCCCTCTGGACCAAAATGAG | CCTTTTCCCACAGGACTTCA |
| *p16* | GCTCAACTACGGTGCAGATTC | GCACGATGTCTTGATGTCCC |
| *Fas* | TGCATGACAGCATCCAAGACA | GCACAGGAGCAGCTGGACTT |
| *Esr1* | ACCATTGACAAGAACCGGAG | CCTGAAGCACCCATTTCATT |

**SUPPLEMENTARY FIGURE LEGENDS**

**Fig. S1 Verification of Lv transduction**

**A** Images of GFP expression (green) of indicated groups.

**Fig. S2 ERα protein levels show no significant difference**

**A** Western blot images with quantification showing ERα protein levels relative to GAPDH from osteoclasts isolated from 8-week-old *Mvp^f/f^* and *Mvp^f/f^Lyz2-Cre* mice following 5 days of osteoclastogenesis induction (n = 3).

**Fig. S3 Verification of OVX and AAV transduction**

**A** The uterine size of indicated groups. **B** Images of GFP expression (green) in distal femurs of indicated groups.

**Fig. S4 Cellular distribution of MVP in human femoral head tissues**

**A** t-SNE visualization of all qualified cells from GSE139396 colored by cell clusters. **B** Expression levels of marker genes in indicated cell clusters. **C** t-SNE visualization colored by cell type annotations. **D** MVP expression levels in indicated cell types.
