# Supplementary figures and images for: Major vault protein (MVP) suppresses aging- and estrogen deficiency-related bone loss through Fas-mediated apoptosis in osteoclasts
Source: Cell Death Dis. 2023 Sep 13;14(9):604. doi: 10.1038/s41419-023-05928-4 (PMC10500014; doi:10.1038/s41419-023-05928-4)

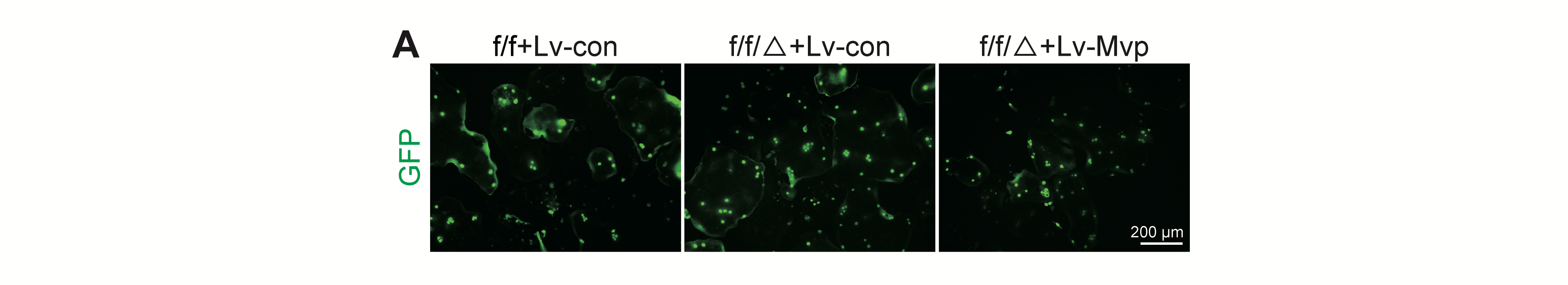

Supplement: Supplementary file 2 — Supplementary Figure 1 [file 41419_2023_5928_MOESM2_ESM.tif]

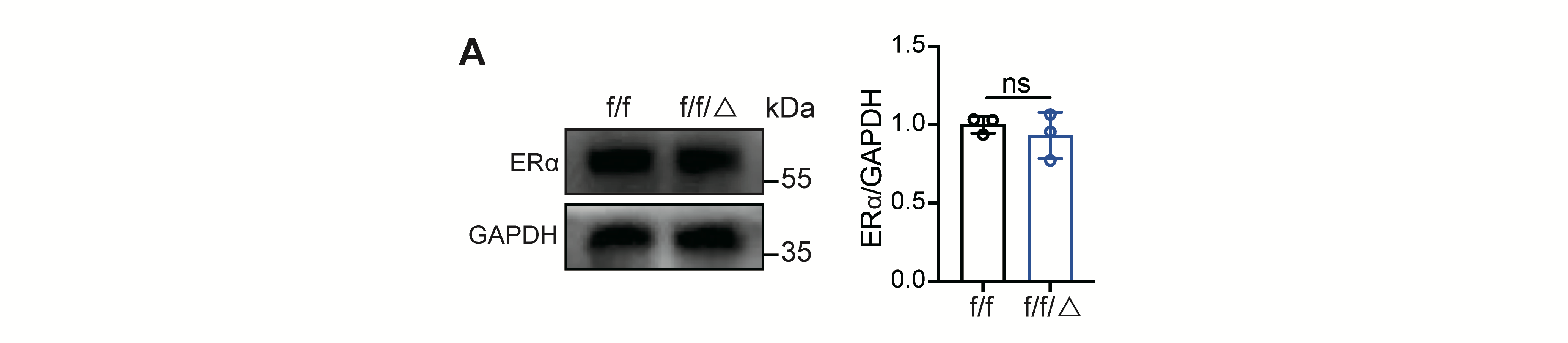

Supplement: Supplementary file 3 — Supplementary Figure 2 [file 41419_2023_5928_MOESM3_ESM.tif]

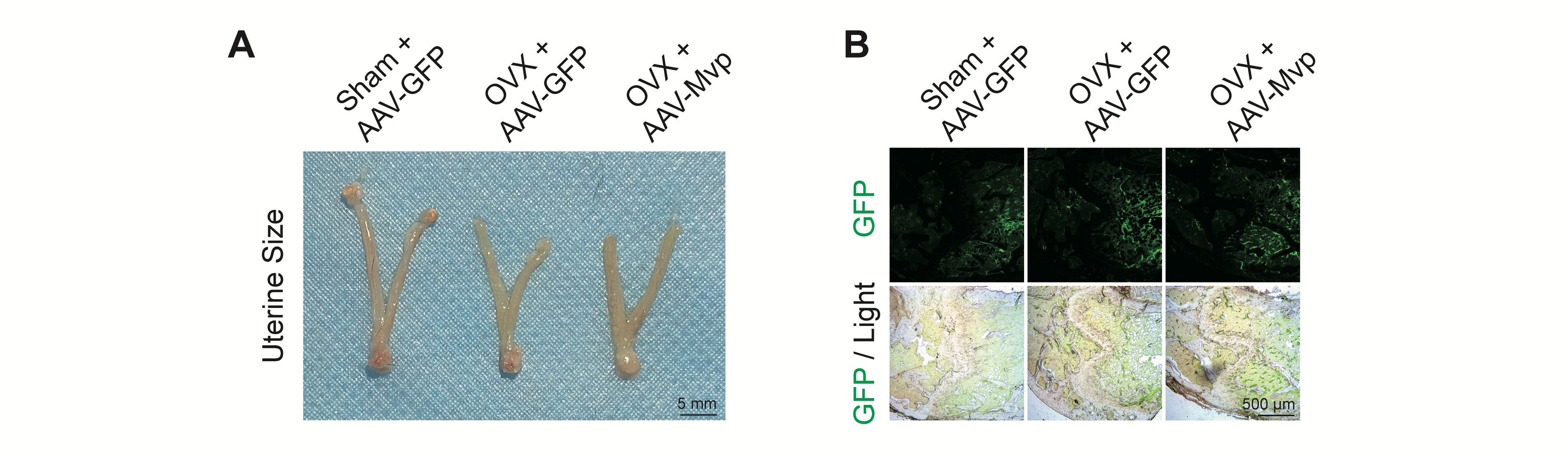

Supplement: Supplementary file 4 — Supplementary Figure 3 [file 41419_2023_5928_MOESM4_ESM.tif]

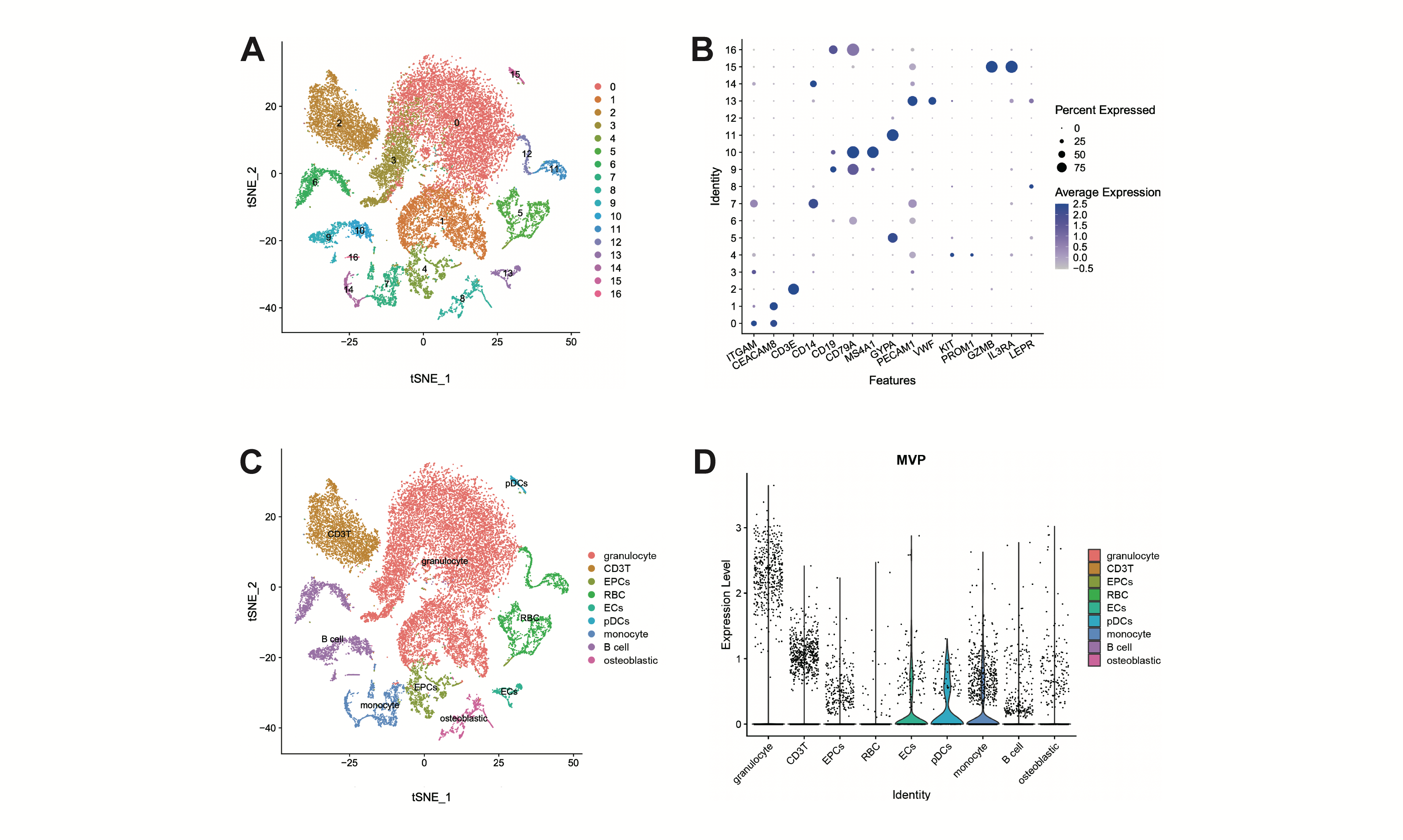

Supplement: Supplementary file 5 — Supplementary Figure 4 [file 41419_2023_5928_MOESM5_ESM.tif]
