# Supplementary material for: Major vault protein (MVP) suppresses aging- and estrogen deficiency-related bone loss through Fas-mediated apoptosis in osteoclasts
Source: Cell Death Dis. 2023 Sep 13;14(9):604. doi: 10.1038/s41419-023-05928-4 (PMC10500014; doi:10.1038/s41419-023-05928-4)

Original Western Blots for Fig. 1B

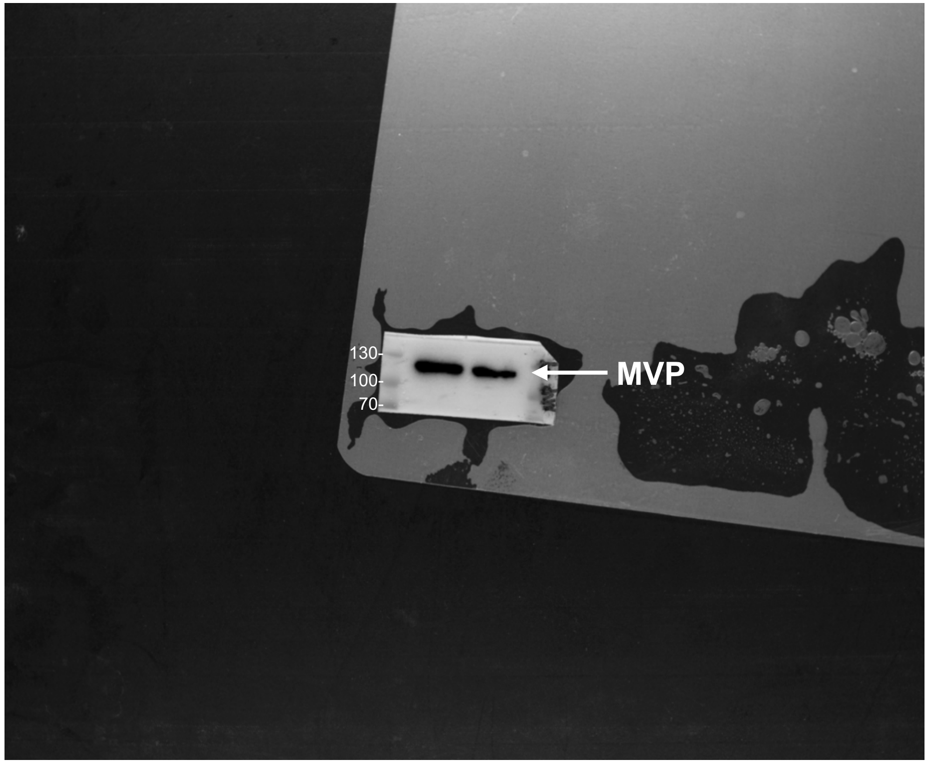

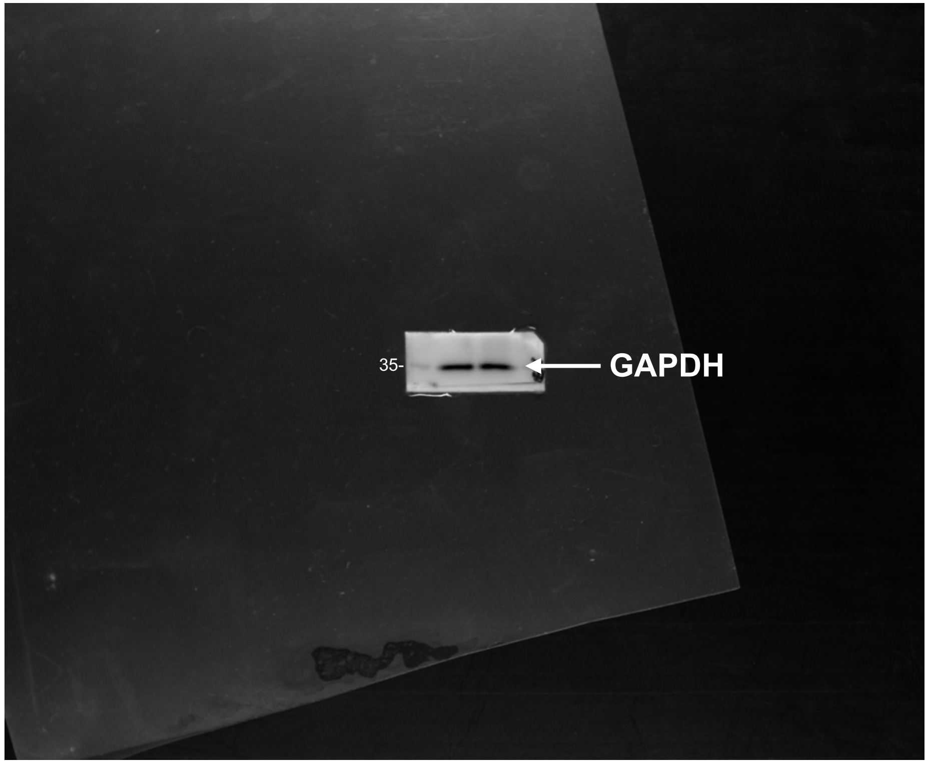

Original Western Blots for Fig. 2A

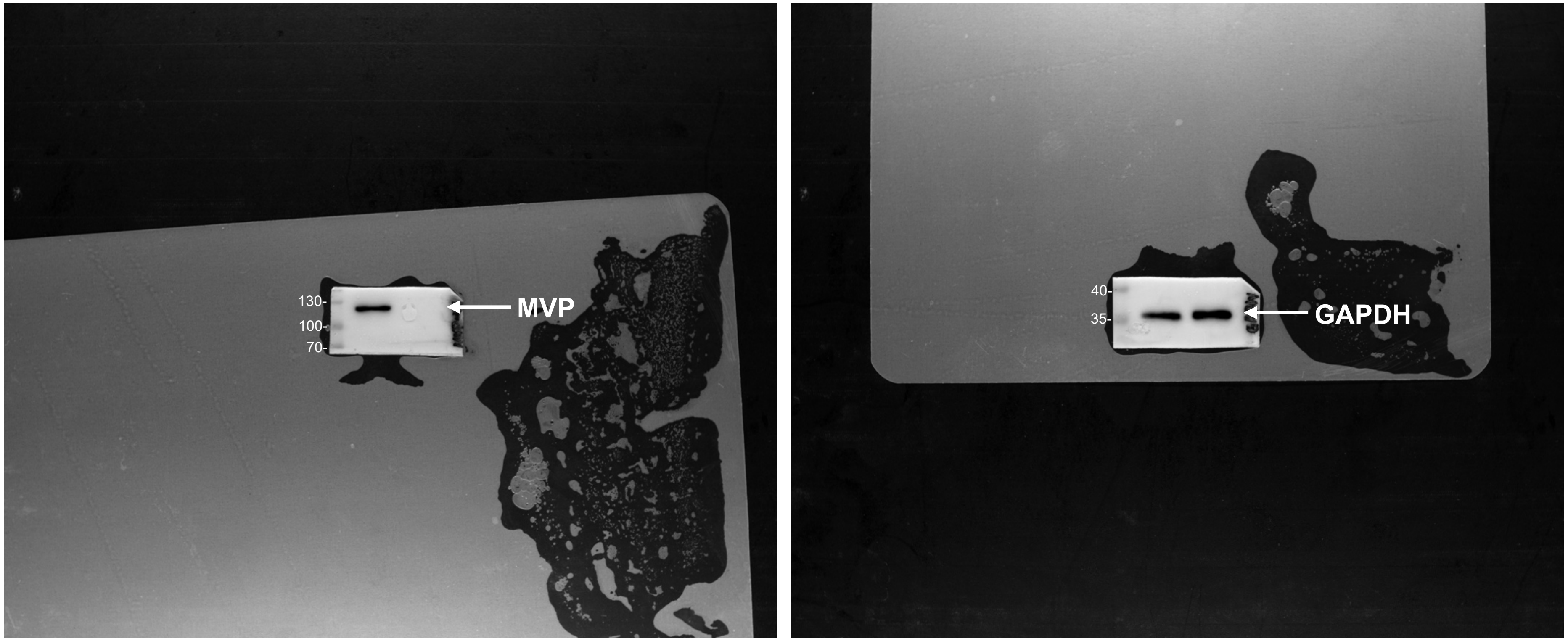

Original Western Blots for Fig. 4C (1/4)

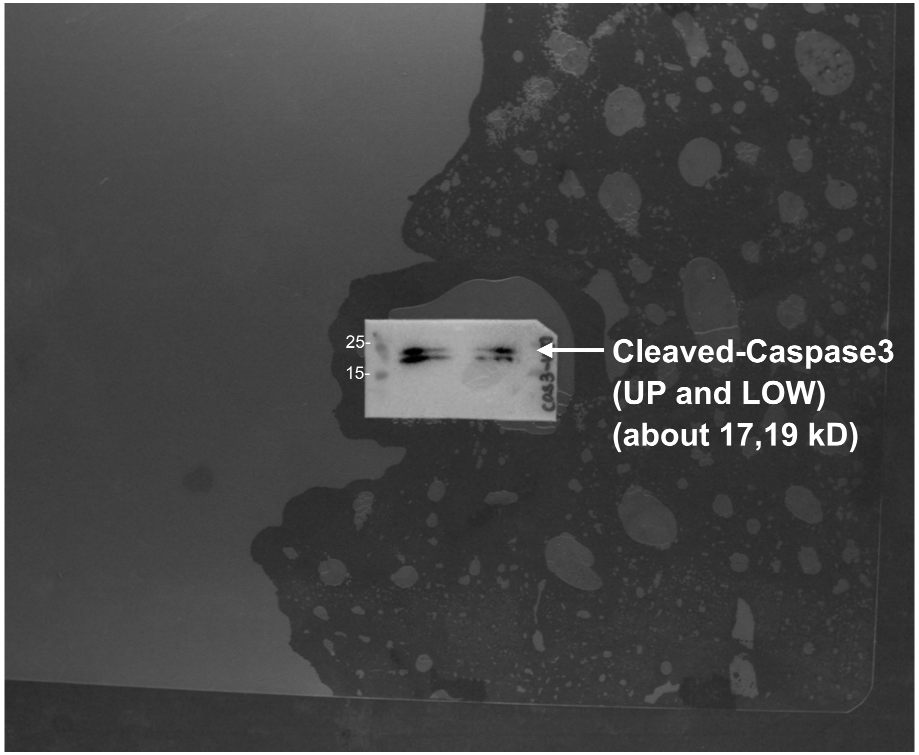

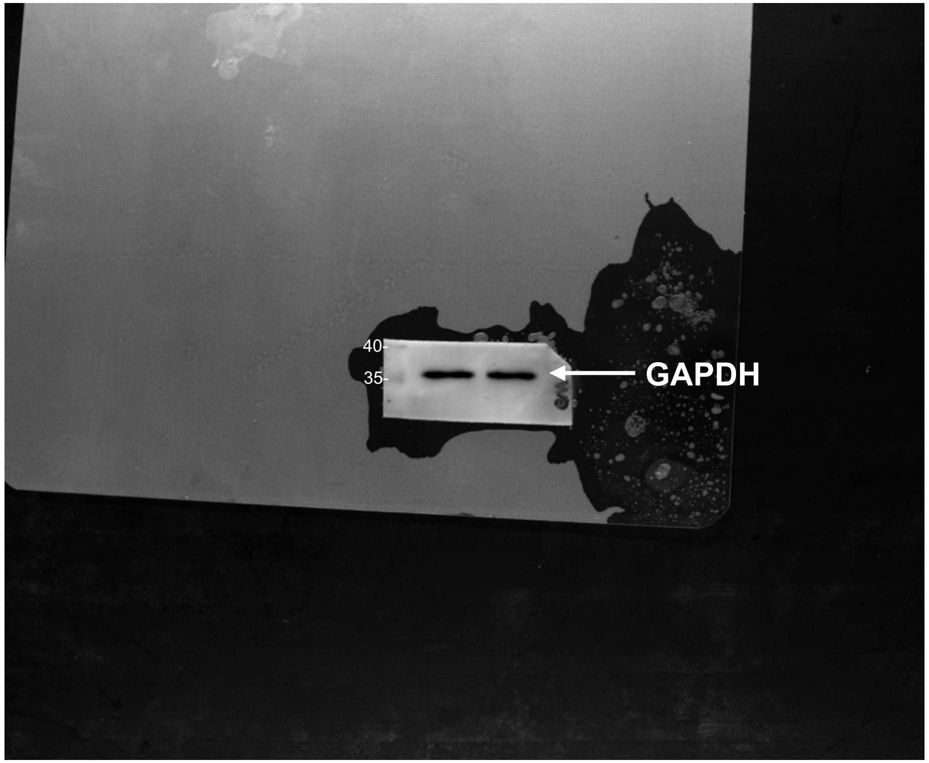

Original Western Blots for Fig. 4C (2/4)

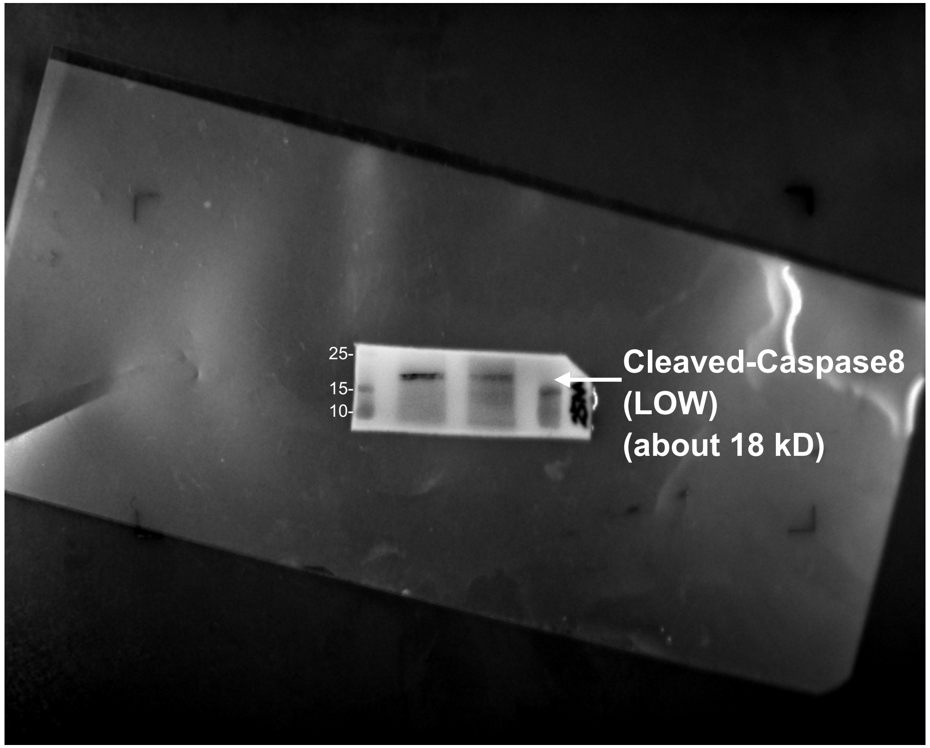

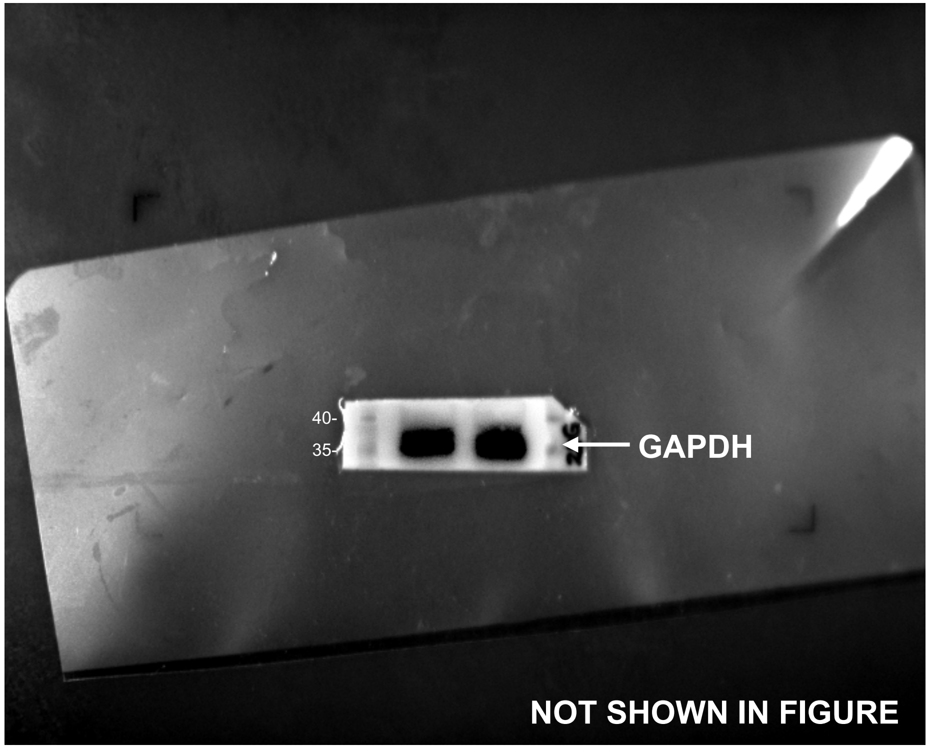

Original Western Blots for Fig. 4C (3/4)

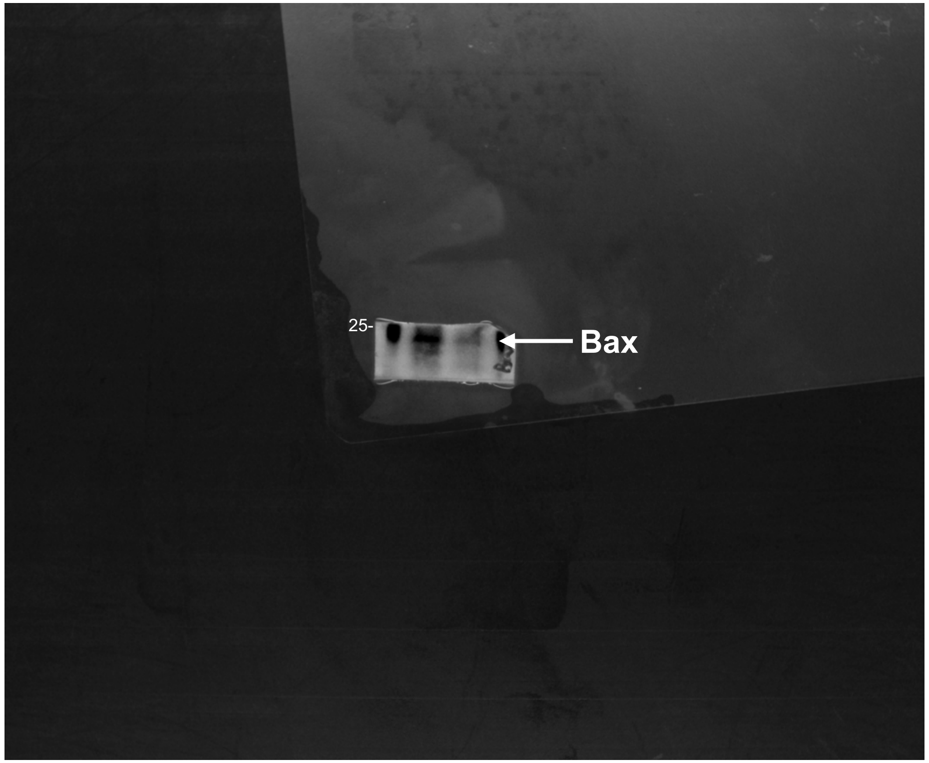

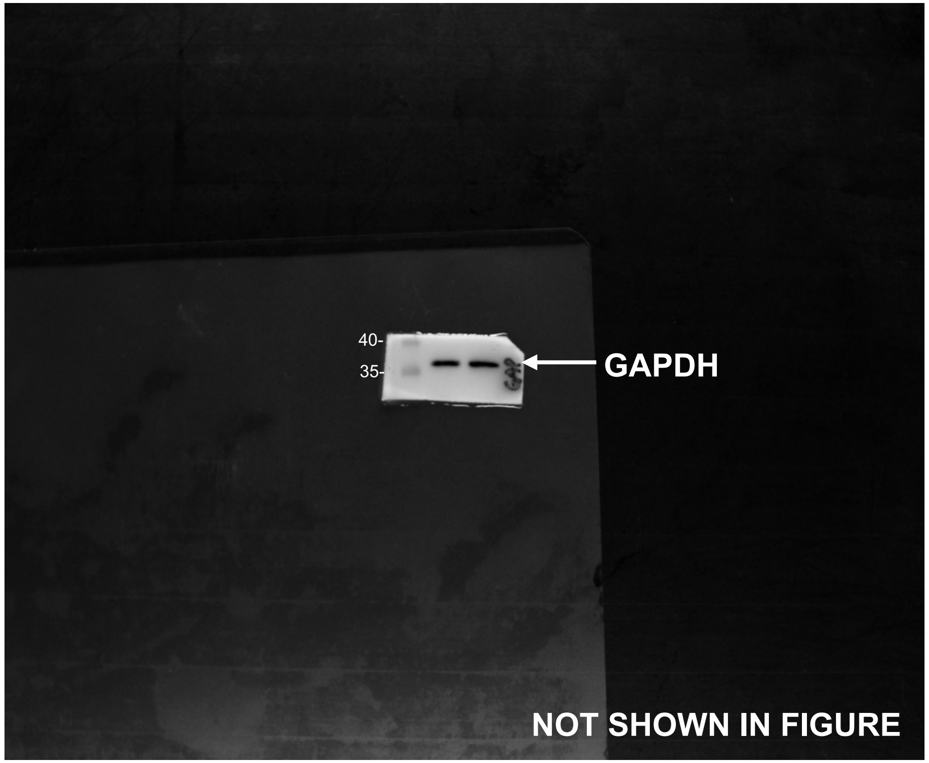

Original Western Blots for Fig. 4C (4/4)

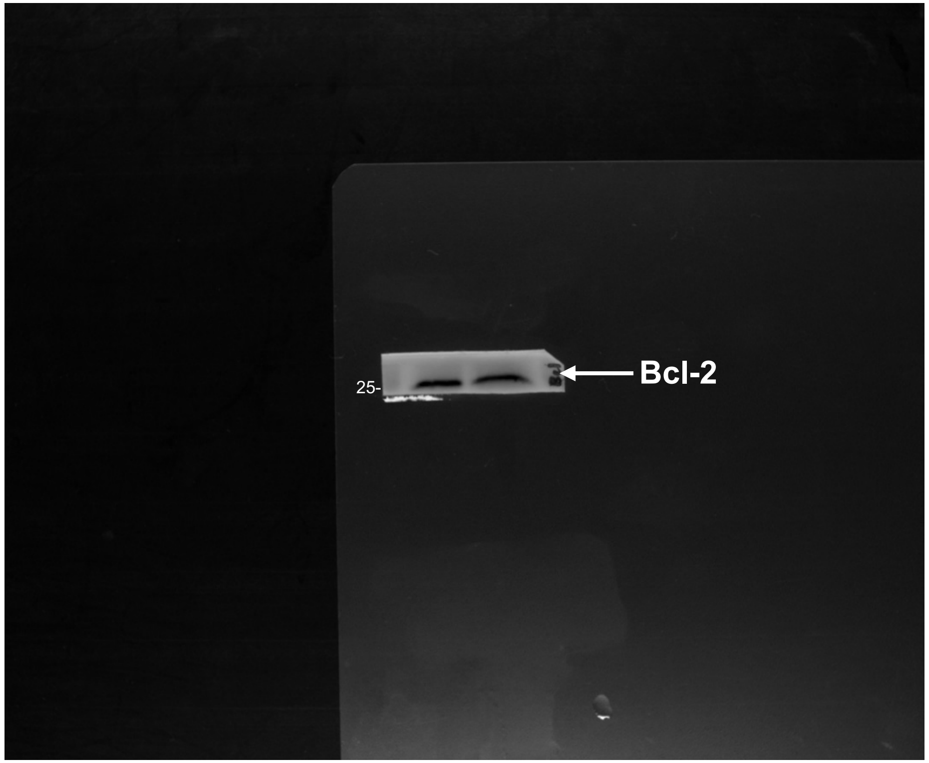

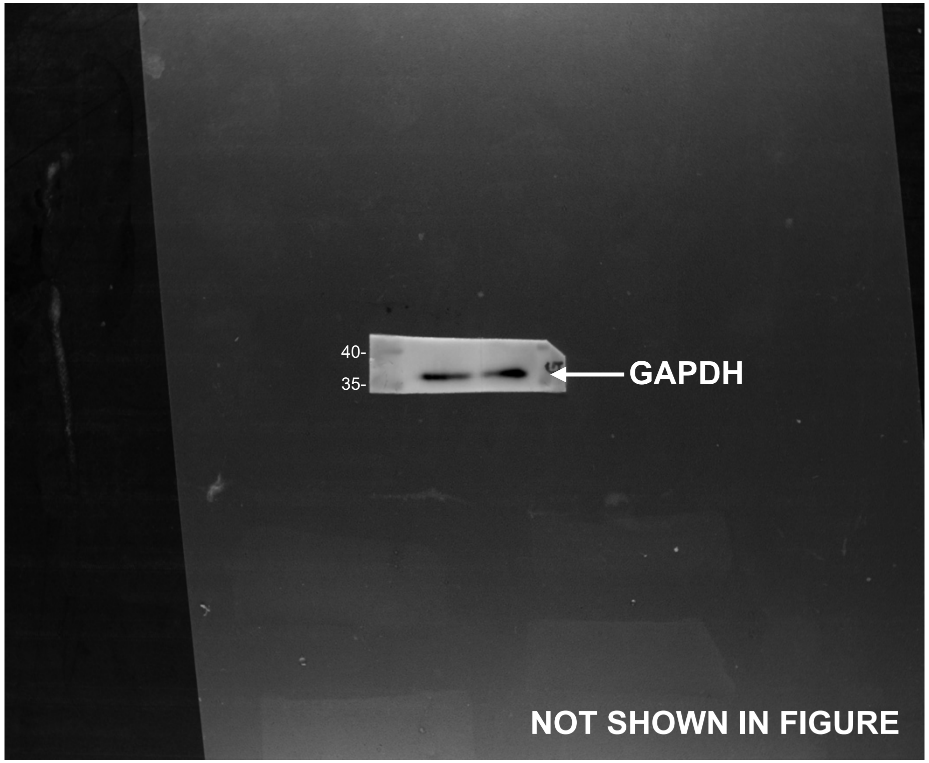

Original Western Blots for Fig. 4D

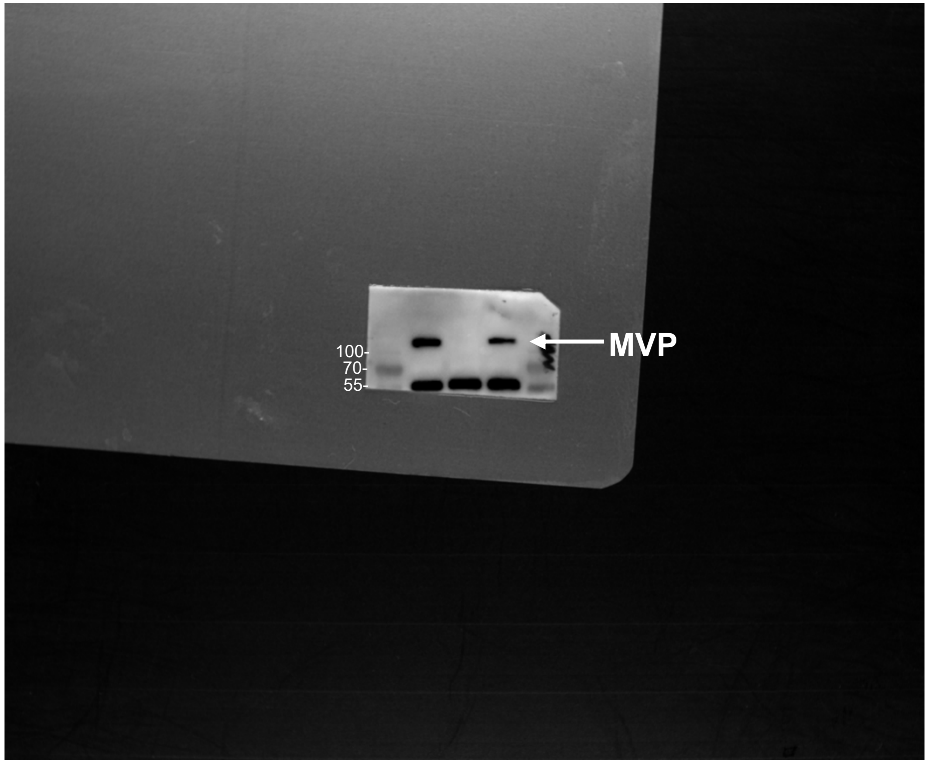

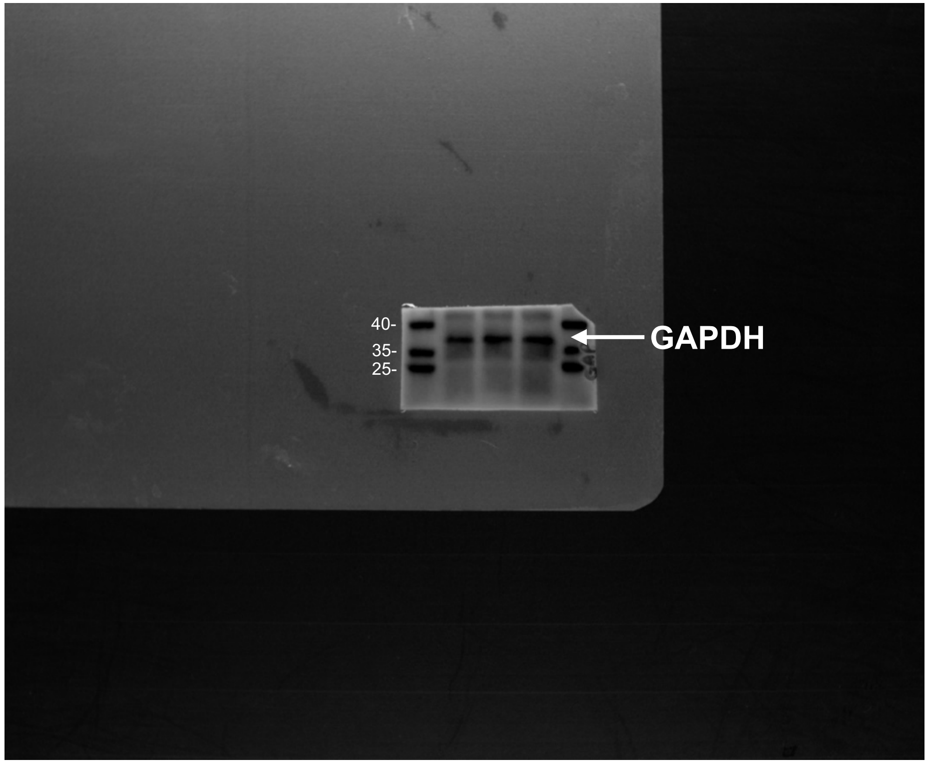

Original Western Blots for Fig. 5A

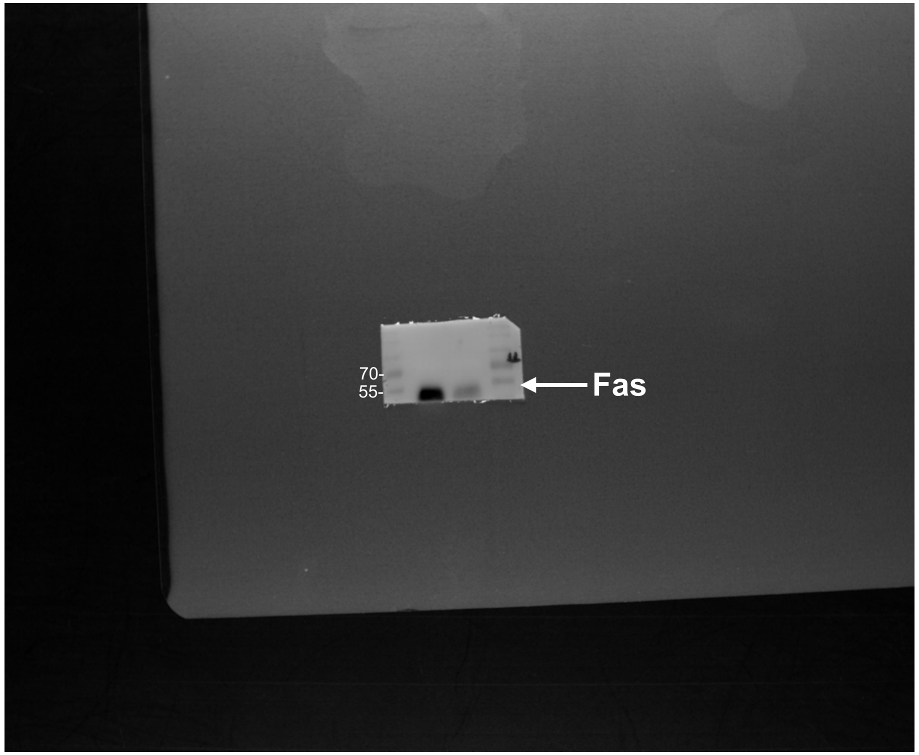

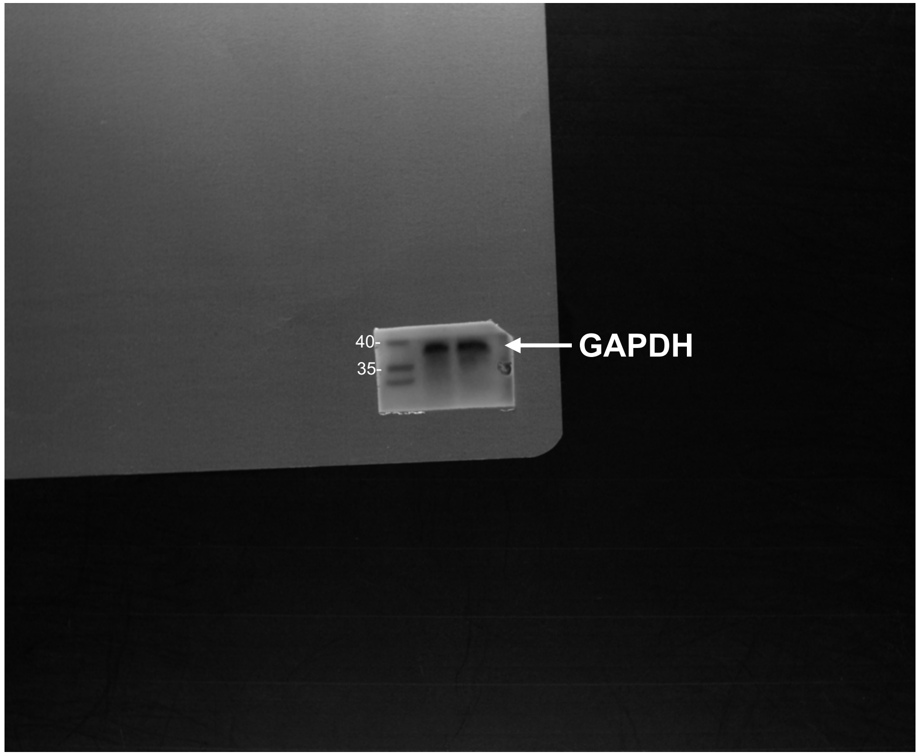

Original Western Blots for Fig. 5D (1/2) IP: Fas

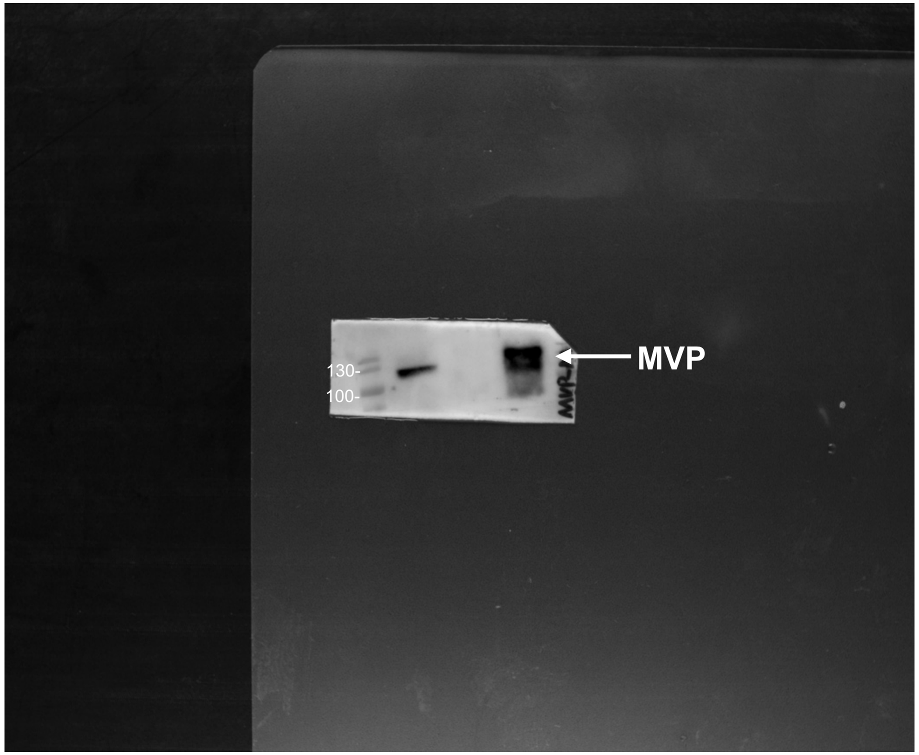

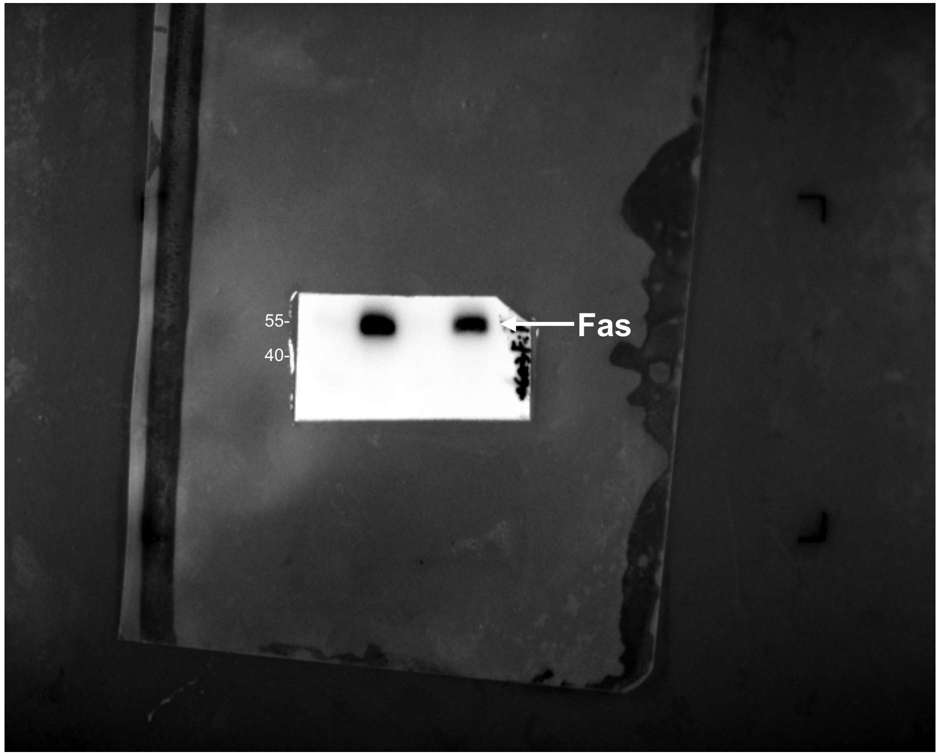

Original Western Blots for Fig. 5D (2/2) IP: MVP

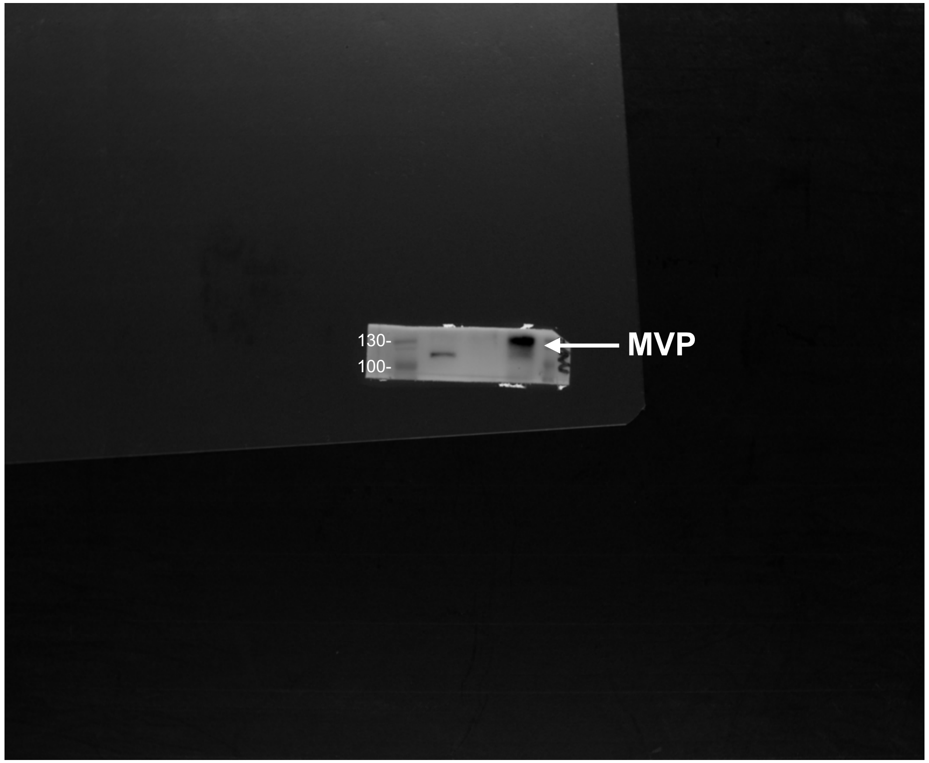

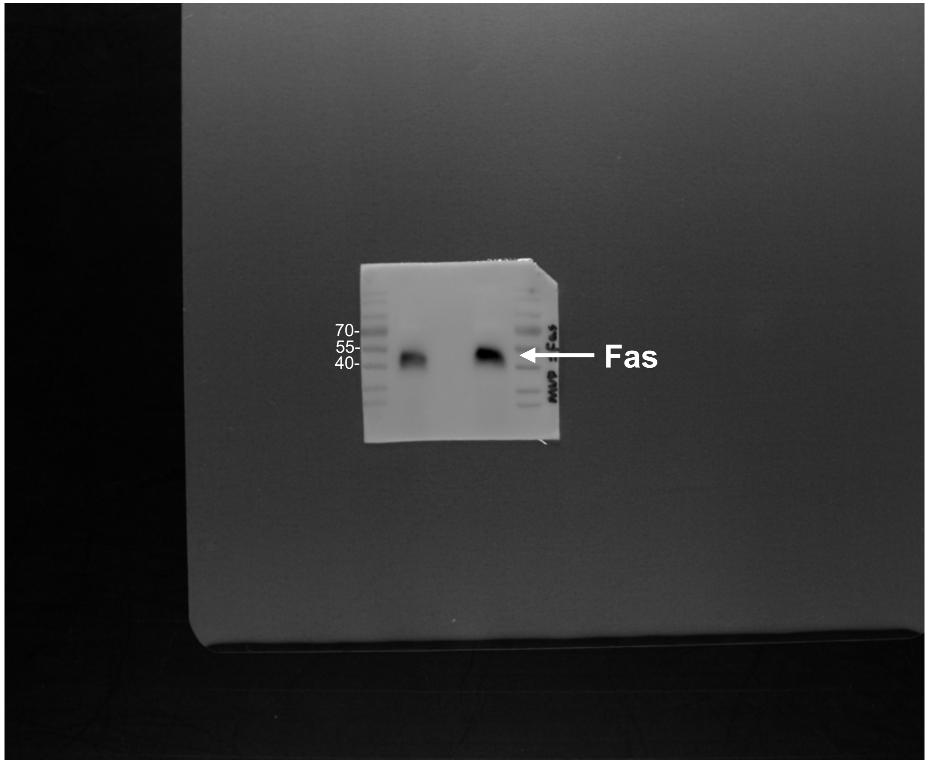

Original Western Blots for Fig. 5F (1/2)

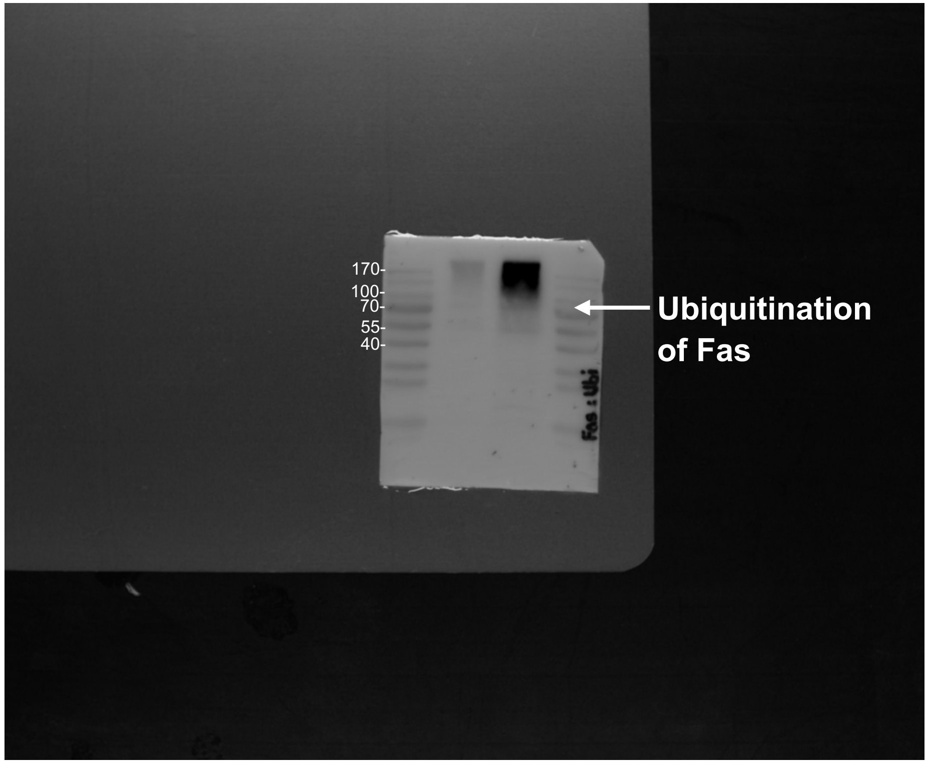

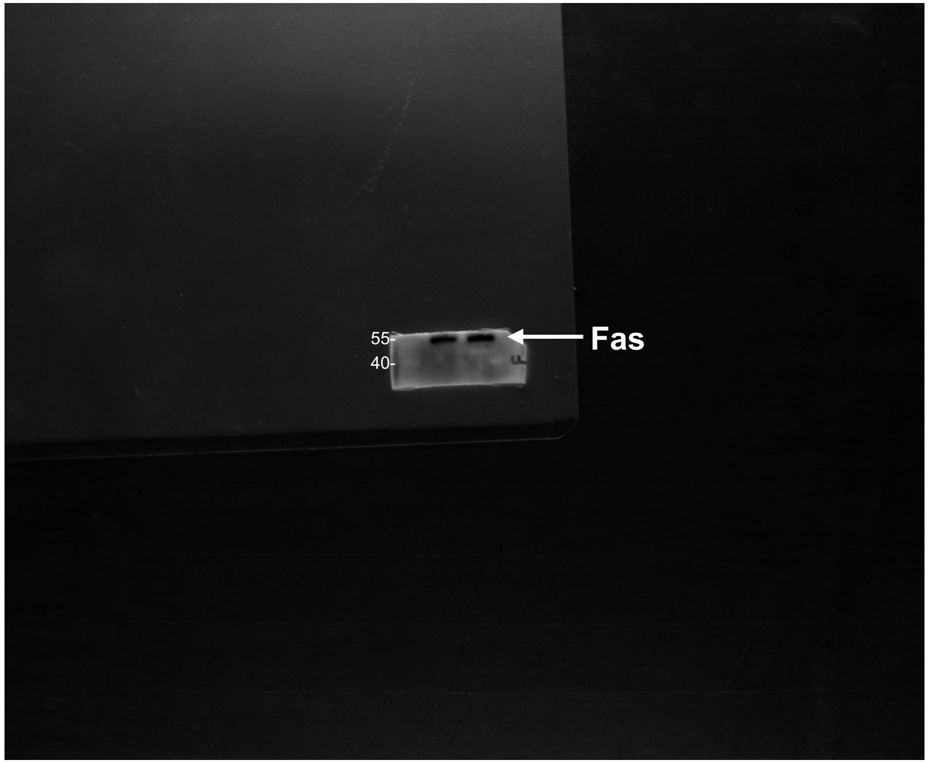

Original Western Blots for Fig. 5F (2/2)

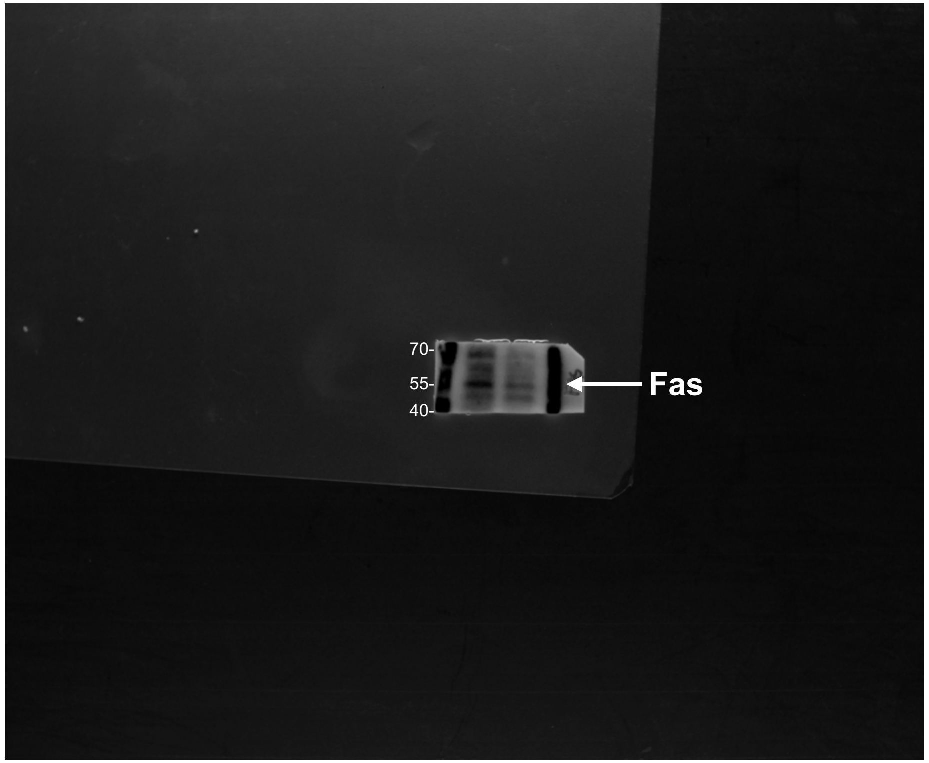

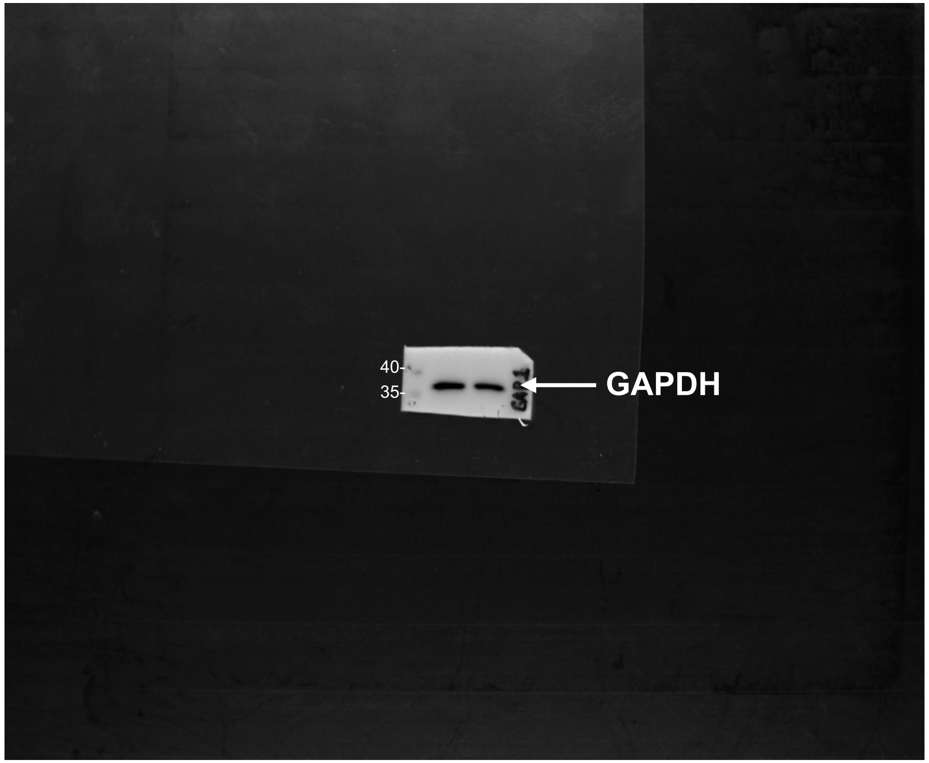

Original Western Blots for Supplementary Fig. 2

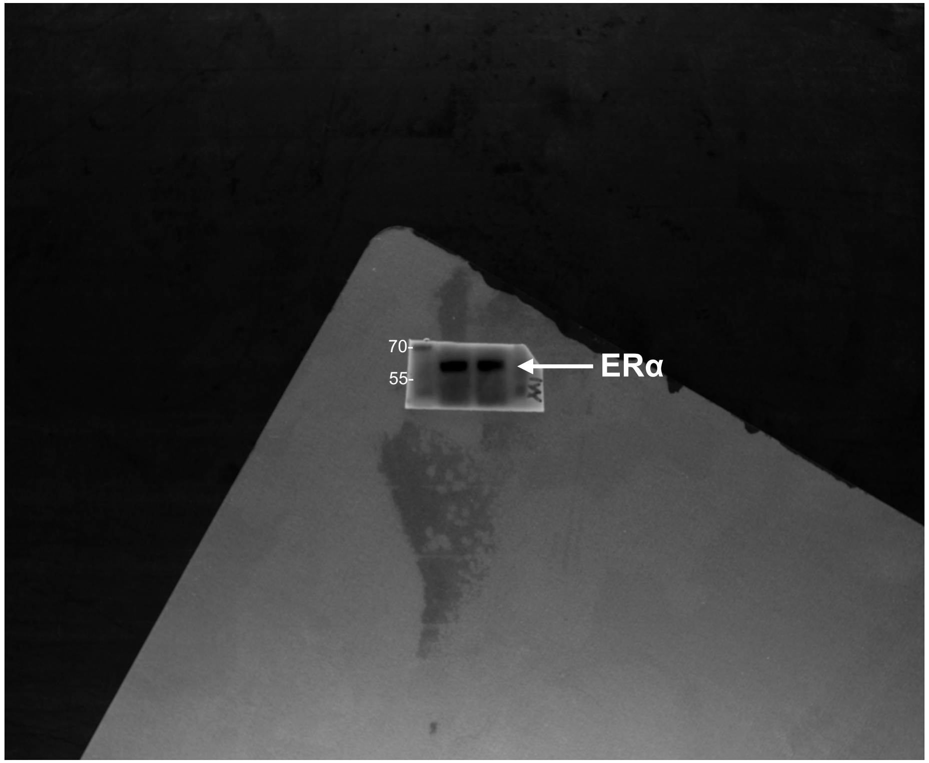

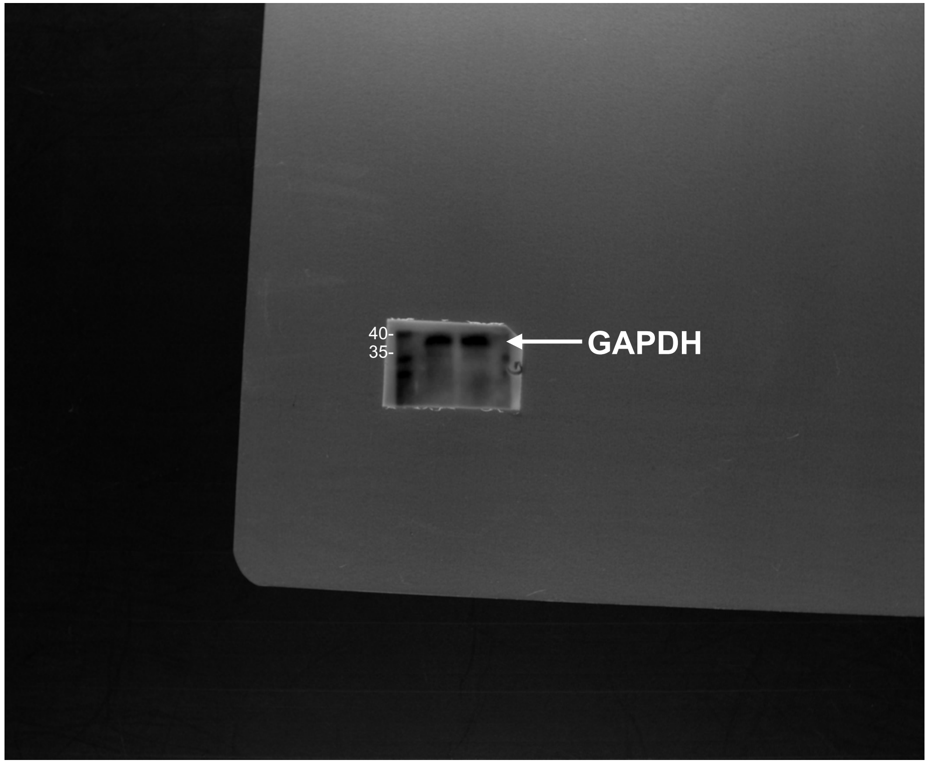

Supplement: Supplementary file 6 — Original Data File [file 41419_2023_5928_MOESM6_ESM.pdf]
